# Show Me What You’ve B/Seen: A Brief History of Depiction

**DOI:** 10.3389/fpsyg.2022.808814

**Published:** 2022-07-11

**Authors:** Inez Beukeleers, Myriam Vermeerbergen

**Affiliations:** ^1^Faculty of Arts, KU Leuven, Leuven, Belgium; ^2^Department of Afrikaans and Dutch, Faculty of Arts and Social Sciences, Stellenbosch University, Stellenbosch, South Africa

**Keywords:** semiotics, depiction, role shifting, constructed action, classifier constructions, depicting sign

## Abstract

Already at a relatively early stage, modern sign language linguistics focused on the representation of (actions, locations, and motions of) referents (1) through the use of the body and its different articulators and (2) through the use of particular handshapes (in combination with an orientation, location, and/or movement). Early terminology for (1) includes *role playing, role shifting*, and *role taking* and for (2) *classifier constructions/predicates* and *verbs of motion and location*. More recently, however, new terms, including *enactment* and *constructed action* for (1) and *depicting signs* for (2) have been introduced. This article provides a brief overview of the history of enactment and depiction in the sign linguistic literature but mainly focuses on issues related to terminology (and terminology shifts). First, we consider the relation between role shifting and constructed action. We question the idea that these terms can be used interchangeably and rather suggest that they capture different, but related functions. Subsequently, we zoom in on the conceptualization of depicting signs, indicating verbs, pointing signs and fully lexical signs and the relation between these signs and the method of depicting. Where earlier research often associates depicting with the use of specific types of structures, we promote the idea that depicting is a semiotic diverse practice. In doing so, we show that the conceptualization of the different sign types and the terms that are used to refer to these phenomena do not accurately capture the way these signs are used in actual signed discourse and propose a reconceptualization of the different sign types in the lexico-grammar of Flemish Sign Language (VGT) as composite signs that can describe, depict and indicate meaning in various ways. In this way, this article illustrates (1) the risks that may come with the execution of terminology shifts and (2) the importance of making a clear distinction between form and function, i.e., we show that it is important to be careful with assuming a (too) exclusive relation between a certain function and one or more particular forms.

## 1. Introduction

Already at a relatively early stage, modern sign language linguistics focused on the representation of (actions, locations, and motions of) referents (1) through the use of the body and its different articulators and (2) through the use of particular handshapes (combined with orientations, locations, and/or motions). Early terminology includes for (1): *role play/playing* (Liddell, 1980; Loew, 1984), *role shift/shifting* (Lentz, 1986; Padden, 1986), *body classifier* (Supalla, 1986), *shifted reference*, *shifted attribution of expressive elements* and *shifted locus* (Engberg-Pedersen, 1993); and for (2): *classifier signs/constructions/predicates* (Frishberg, 1975; Kegl and Wilbur, 1976; Supalla, 1986), but also *verbs of motion and location* (Supalla, 1978), *spatial-locative predicates* (Liddell and Johnson, 1987), *polymorphemic verbs/predicates* (Wallin, 1990; Engberg-Pedersen, 1993), *productive signs* (Brennan, 1992) and *polysynthetic signs* (Takkinen, 1996). As becomes also clear when reviewing the terminology uses, these constructions have been conceptualized as symbolic, i.e., morphologic structures that encode linguistic meaning. More recently, and partly as a result of the rise of Cognitive Linguistics and the increasingly closer links between Signed Language Linguistics and Gesture Studies, new terms have been introduced. The representation of referents through the use of the own body is now also referred to with, for instance, *constructed action* and *enactment/enacting* and the term *depicting signs* was introduced to refer to *classifier constructions*. Evidently there is more to it than simply a new name: many researchers have moved away from the symbolic, morphologic conceptualization of classifier constructions and role shifts and rather suggest that these constructions are (partly) gestural in nature. In doing so, they promote the idea that gesture is an integral part of language (see for instance, Liddell, 2003 for ASL; Vermeerbergen and Demey, 2007 for VGT; Ferrara, 2012; Johnston, 2013; Ferrara and Hodge, 2018 for Auslan, amongst many others, but see – for instance – Garcia and Sallandre, 2020 for a Semiological approach and Lepic and Occhino, 2018 for a Construction Morphology approach).

In this article, we first provide a brief history of the conceptualization of classifier constructions and role shifting, with a particular focus on the terminology. Subsequently, we highlight that adopting new terminology does not always come without risks. When reviewing literature on role shifting, for example, it becomes apparent that it often remains unclear how older and newer terms relate to one another and whether these terms refer to the same concept or function. In part 2 we therefore go into the relation between role shifting and constructed action. In more recent literature, we regularly find statements such as *constructed action, previously known as role shift*. In this article, however, we suggest that these different terms are possibly being used to capture and describe (slightly) different functions in signed language discourse. In part 3, we continue on this line by focusing on the conceptualization of classifier constructions (or depicting signs)^1^, indicating verbs and pointing signs, and on the relation between these structures and the method of depicting, i.e., showing. Where researchers traditionally have emphasized a strong relation between depicting and the use of classifier constructions and constructed action, we show that signers have various types of semiotic signs at their disposal when they want to depict meaning. We also question the idea that the main function of a classifier construction or a stretch of enactment is always depiction and we argue that signers can also use these highly iconic structures to mainly describe meaning. In doing so, we show that the theoretical conceptualization of these signs and the terminology used to refer to these mechanisms can be misleading and do not accurately capture how these signs are used in actual signed discourse. In this way, we also show that assuming a (too) exclusive relation between a certain function and a particular form can be problematic. Finally, in part 4 we reflect on the implications of this contribution and put forward some suggestions for future research.

## 2. The Study of Role Shifting and Classifier Constructions: Now and Then

In this first part of the paper, we provide an overview of literature on role shifting/role playing and classifier constructions. Although this review is not exhaustive, it highlights the most important theoretical evolutions, with concomitant terminology shifts, in the field of Signed Language Linguistics and it will provide the necessary theoretical background for parts 2 and 3 of the paper.

### 2.1. “Role Playing”

Already in the 1970s researchers working on American Sign Language discuss the use of the signer’s body to refer to somebody else. According to Friedman (1975) for example, American signers may use their body to mark third person (3P) instead of making an indexic reference. The signer is said to ‘‘take on’’ a third person reference, ‘‘in much the same way (conceptually) that the speaker takes on 3P reference in 3P narrative prose in oral language’’ (1975:950). Friedman points out that this process, i.e., conveying 3P reference by the use of surface 1P forms, is very common in ASL^2^ and is most clearly seen when the discourse concerns more than one 3P referent. She presents an example of a mother-child interaction and points out that the signer not only uses the body and/or head to distinguish between the two referents but that he will also “look up (with his head raised) when he assumes the child’s role, and will look down (with his head lowered) when he assumes the mother’s role” (1975:950).

In publications from the 1970s to 1980s, role playing/role taking/role shifting was mainly associated with reported speech/direct quotation. Somewhat generalized, this is how it was presented: Like speakers, signers can opt for direct quotation/reported speech to report what someone else said/signed. This implies that the speaker/signer “shifts” in the role of the original speaker/signer and makes the report from the quoted person’s point of view. Such role shifting usually involves a shift in body position, facial expression and/or eye-gaze (e.g., Mandel, 1977; Padden, 1986 for ASL). This is then usually illustrated with an example of a dialogue between two people, where “a signer may alternate roles to speak each person’s lines in turn, taking one role by shifting his stance (or just his head) slightly to the right and facing slightly leftward (thus representing that person as being on the right in the conversation), and taking the other role by the reverse position” (Mandel, 1977, p. 79). Within the at that time dominant formalist perspective, authors mainly focused on the morphosyntax of the direct quotation and discussed, e.g., agreement markers in verb agreement and the interpretation of pronouns and indexicals.

Liddell (1980), in a chapter on non-manual signals in ASL, introduces “what has been called ‘role playing.”’ That is, in his example (15) the speaker adopts someone else’s point of view when he does this (1980: 25). According to Liddell (1980, p. 25) the following sequence means that Bill *decided* that some other person went to the movies^3^:


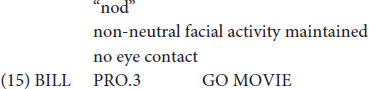


This example is highly similar to Liddell’s example (16) on p. 26, the only difference being that PRO.3 (third person reference) is replaced with PRO.1 (first person reference). In contrast, however, the latter sentence (with PRO.1) means that Bill decided that *he himself* would go to the movies. This is an interesting example also because it is not a case of reported *speech* in the strict sense, i.e., it is not about what Bill *said/signed* but about what Bill *decided*/*thought*. Liddell (1980) discusses another example of role playing where the signer takes on the role of a dog. Here “a signer signed DOG, looked from side to side as if checking to see if the coast was clear and then signed FINE, meaning the dog thought, ‘fine!’ (…) (Signed with other non-manual behaviors the sequence could mean, ‘The dog is fine’)” (Liddell, 1980, p. 56). Liddell refers to Bendixen (1975) who differentiates between the pantomimic “role establishment” and the subsequent “role playing.” It is not fully clear to us whether Liddell himself agrees with the analysis that the pantomimic behavior following the sign DOG and preceding the sign FINE is not part of the role playing, as he claims that “adopting a role is common in stories and everyday conversation. It can be used for direct quotation or for the pantomimic reenactment of an event” (Liddell, 1980, p. 56, emphasis added).

Despite the frequent linking of role shifting and direct quotation, some of the early publications thus already noted that it is not only about representing the *speech* of a referent but also about thoughts, feelings and (for some authors) actions. Engberg-Pedersen (1993, for DTS), for instance, argues that role shifting is not a sufficiently accurate term, because signers may use the sender locus to refer to one referent, while simultaneously expressing the emotions of another referent. According to Engberg-Pedersen (1993, p. 103), it is important to distinguish between three different functions, i.e.,

• 1. Shifted reference, i.e., the use of pronouns from the quoted sender’s point of view, especially the use of the first pronoun 1. p to refer to somebody other than the quoting sender,
• 2. Shifted attribution of expressive elements, i.e., the use of the signer’s face and/or body posture to express the emotions or attitude of somebody other than the sender in the context of utterance,
• 3. Shifted locus, i.e., the use of the sender locus for somebody other than the signer or the use of another locus than the locus c for the signer.

Engberg-Pedersen (1993) claims that whereas shifted reference is restricted to reported speech, the other two phenomena can also occur in other types of signing. Building on Tannen (1986), she also indicates that shifted attribution of expressive elements in Danish Sign Language is comparable with the way speakers can change their voice in order to take on characters’ voice.

Within the framework of his semiologic model, Cuxac (1985, 2000) distinguishes between two modes of communication: telling without showing (*dire sans montrer*) and telling by showing (*dire en montrant*). Cuxac (1985, 2000) takes the signer’s intention as a starting point and argues that if signers want to tell by showing, they can make visible the real life or imaginary experiences and observations through the use of highly iconic structures or transfers. One of these transfers considers the personal transfer (transfert personnel), by which the signer literally takes on the role of the entity he refers to Cuxac (2000, p. 51) proposes the following characterization:

*Ces structures reproduisent, en mettant en jeu tout le corps du locuteur, une ou plusieurs actions effectuées ou subies par un actant du procès de l’énoncé: humain ou animal le plus fréquemment, mais ce peuvent être aussi des non-animés […]. Le narrateur “devient,” pour ainsi dire, la personne dont il parle, jusqu’à, chez certains locuteurs, lui ressembler physiquement. Pour caractériser ces structures, les Sourds utilisent un signe de leur langue signifiant approximativement “rôle” ou “prise de role.”*

(*These structures reproduce, by bringing into play the whole body of the speaker, one or several actions carried out or undergone by an actant of the process: human or animal most frequently, but they can also be non-animate [.]. The narrator “*becomes,” *so to speak, the person he is talking about, to the point where, for some speakers, he resembles him physically. To characterize these structures, the Deaf use a sign in their language that roughly signifies “role” or “role-taking”*).

The idea that signers want to show (i.e., depict) actions when physically embodying a referent is also present in literature on American Sign Language. In contrast with Cuxac (1985, 2000), however, the use of the own body to depict actions, thoughts or feelings is not conceived as entirely linguistic. When conceptualizing the phenomenon researchers rather also start to consider the role of gesture in signed languages. This becomes, for instance, apparent with the introduction of the notions of *constructed action* and *constructed dialogue* (Winston, 1991, 1992; Metzger, 1995; Liddell and Metzger, 1998). Winston (1991, p. 404), who was inspired by Tannen (1986), considers constructed dialogue as one type of *performative*, i.e., it “shows the actions and persona of the speaker.” Performatives include both constructed dialogue and *performance of an action* (1991:400) or *action performatives* (1992:100). In a similar vein, Metzger (1995) suggests that constructed dialogue is one of the various types of constructed action occurring within the discourse. She sees constructed action as a discourse strategy and describes it as “the creative construction of an event described by a signer in ASL discourse” (1995:266).

Liddell and Metzger (1998) and Liddell (2003) conceptualize constructed action within Fauconnier’s (1985) Mental Space Theory and the notion of mental space blends (Fauconnier and Turner, 1996). In doing so, they argue that signers can bring referents into real space, i.e., their immediate environment, through the process of blending. When constructing action, signers blend the referent performing the action onto their own body. “Within the context of the blend, the actions of the signer demonstrate the attributes of the character blended with the signer” (Liddell and Metzger, 1998, p. 676). They also suggest that constructed action is gestural in nature and compare this phenomenon with McNeill’s (1992, p. 12–14) iconic gestures, i.e., gestures that accompany speech and illustrate concrete actions [see also, for instance, Emmorey (1999, p. 145) on the comparison of constructed action with Clark’s (1996) component iconic gestures]. Such approaches illustrate some important developments in the field of Signed Language Linguistics, and in the field of linguistics more generally, i.e., with the rise of Cognitive Linguistics and the introduction of Gesture Studies into the discipline of Linguistics a multimodal and multi-semiotic view of human (spoken and signed languages) was promoted (Garcia and Sallandre, 2020).

Soon after Metzger’s introduction of the term ‘‘constructed action’’ in 1995, more and more researchers started to use this notion, often providing their own definition. Some examples are as follows^4^:

– “Gestures intended to illustrate the actions of others” (Liddell and Metzger, 1998, p. 660).
– “Constructed action refers to the gestures that imitate the actions of someone other than the signer at the time of signing” (Johnston and Schembri, 2007, p. 273).
– “Becoming an object”; “the use of the signer’s body to depict the actions and movements of an object – whether that object be animate or inanimate” (Quinto-Pozos, 2007, p. 1285).
– “Constructed action thus refers to those gestures and bodily behaviors that are used either (i) at the same time as signing or (ii) instead of signing” Johnston (2013, p. 53).
– “Constructed action (CA, or enactment, also known as role-shift), where the signer uses his or her body (the head, face, arms, and torso) to represent the thoughts, feelings or actions of a referent using the surrounding space on a real world scale” (Cormier et al., 2013, p. 370).
– “That Constructed Action is a stretch of discourse (however, short or long) that represents one role or combination of roles depicting actions, utterances, thoughts, attitudes and/or feelings of referents other than the signer (narrator)” (Cormier et al., 2015, p. 195).

Finally, and also in light of the rise of Gesture Studies, *constructed action* is also often used interchangeably with *enacting gestures* and *enactment*, with the understanding that “*enactment*” includes both constructed dialogue and constructed action (in the narrow sense) (see for instance, Ferrara, 2012; Hodge and Ferrara, 2014). Ferrara (2012, p. 64) defines enacting gestures as “gestures that do (partially) demonstrate actions or events.” In a more recent publication, Ferrara and Halvorsen (2017, p. 376) write:

“Another type of iconic, semiotic action available to speakers (and as we shall see, signers) in their multi-modal communicative repertoire is the practice of demonstrating the thoughts, words, or actions of (real or imagined) referents. These enactments occur when a person recruits any number of articulators, e.g., each of the hands, arms, head, face, shoulders, torso, vocal tract, etc., to produce iconic demonstrations.”

From the beginning, there was confusion as to how exactly the term *role-shifting* was understood. Padden (1986) for example, writes: “In a role-shifting structure, third person pronouns are shifted into first person. Role-shifting is marked by a perceptible shift in body position from neutral position (straight facing) to one side and a change in direction of eye gaze for the duration of the *role*” (1986:48). For Padden (1986), body shift and the shifted eye-gaze are ways of marking role shifting rather than (part of) the role shifting itself. For other authors, body posture and eye-gaze do seem to be part of the role-shift and/or changes in body posture are equated with role-shift. The same vagueness can also be found in descriptions of constructed action and enactment. Sometimes these terms seem to concern the specific behaviors/activities of the signers, while elsewhere those behaviors/activities are presented as ways of expressing CA/enactment. Yet other researchers approach CA or enactment more as *stretches of discourse* or as a certain *behavior*.

In the current Signed Language Linguistics literature, (1) *role-shifting*/*role-taking*, (2) *constructed action*/*constructed dialogue* as well as (3) *enactment* are used, sometimes with the same meaning, sometimes not. As shown above, this variety of terms, over the years but also today, is undoubtedly related to different (theoretical) approaches and interpretations. However, the multiplicity of terms also indicates the great complexity of the phenomenon (or phenomena). Cormier et al. (2015, p. 169) note:

“Terminology used to refer to this phenomenon varies considerably, and it is often unclear if the same assumptions about its nature are being made by different researchers. It is often not even clear whether these terms are used to refer to the same phenomenon, different aspects of the same phenomenon, or perhaps different phenomena altogether.”

In the following sections, we follow up on the ambiguous relation between the concepts of role shifting on the one hand, and constructed action on the other hand. We investigate the relation between the two terms and the concepts they refer to.

### 2.2. Classifier Constructions

#### 2.2.1. Formalist, Morphemic Approaches: Classifiers

Already in early studies on signed language structures, researchers noted the existence of complex predicates that express a referent’s movement and/or location. These constructions have been analyzed in various ways, which in turn has also led to a variety of terms used to identify them, including *classifier*, *classifier predicates*, or *classifier constructions* (e.g., Frishberg, 1975; Kegl and Schley, 1986; Schick, 1990 for ASL; Vermeerbergen, 1996 for VGT), *spatially descriptive signs* (DeMatteo, 1977 for ASL), *verbs of motion and location* (Supalla, 1978, 1990 for ASL), *polymorphemic verbs* (Engberg-Pedersen, 1993 for DTS), *polysynthetic signs* (Wallin, 1990 for SSL), *productive lexicon/productive signs* (Brennan, 1990 for BSL; Vermeerbergen, 1996 for VGT) *depicting verbs* (Liddell, 2003; Dudis, 2004 for ASL), and *depicting signs* (Johnston and Schembri, 2007; Ferrara, 2012 for Auslan). In some of the early studies, these constructions were described as a form of visual imagery, i.e., as non-morphemic, complex, iconic constructions that express referent’s movements’ and locations (DeMatteo, 1977 for ASL).

However, soon after the start of modern Signed Language Linguistics more formalist, morphemic conceptualizations became dominant. Supalla (1978), for instance, argues that these classifier constructions (or in his words: verbs of motion and location) consist of multiple movement roots combined with movement affixes and possibly also articulator affixes. The movement roots express the existence, location or movement of the noun. Signers can combine these movement roots with movement affixes in order to refer to the manner of movement. In doing so, they can for instance indicate a bouncing movement or a circle path movement. Finally, the articulator morpheme in this model refers to (part of) the signer’s hand, which classifies characteristics of the noun referent in the construction, i.e., of the referent that is being located/is moving. In this study, and in other studies that adopt a more formal perspective to the phenomenon, it becomes apparent that the iconic nature of these constructions is downplayed or even ignored. The focus in these studies is rather on the comparability of these classifier constructions with classifiers in spoken languages (see also Schembri, 2003).

#### 2.2.2. Cognitive, Functional Approaches: Morphemic, Yet Iconic Constructions

Engberg-Pedersen (1993 for DTS) approaches classifier constructions, which she refers to with the term *polymorphemic verbs*, from a cognitive, functional point of view. Just like Supalla (1978), she conceptualizes these constructions as morphemic. According to her model, signers combine the verb stem (i.e., the handshape) sequentially and/or simultaneously with multiple movement morphemes, which can express the location, motion, distribution, manner, extension, and/or aspect. Note, however, that there are a couple of important differences in this analysis compared to most of the formalist approaches, including Supalla (1978). First, Engberg-Pedersen (1993) argues that the handshape is the verbs stem, rather than the movement roots (e.g., Supalla, 1978). Second, Engberg-Pedersen (1993) also explicitly argues against the idea that the handshapes (i.e., often referred to as classifiers) are comparable to classificatory verb stems in some spoken languages, like Athapaskan languages. Finally, she also acknowledges the iconic nature of these constructions. In her analysis of the handshape stems, for instance, she distinguishes between different types of stems based on their iconic origins. Whereas whole entity stems iconically represent the entire referent, limb stems refer to the entire referent by presenting its limbs. Moreover, Engberg-Pedersen (1993) also argues that the movement of classifier constructions is often also an iconic rendition of the movement of the referent in the scene talked about. This rediscovery of the iconic nature of classifier constructions becomes also apparent in the work from other cognitive and functional linguists (see also, for instance, Schick, 1990 for ASL; Brennan, 1990 for BSL; Cuxac, 1996, 2000 for LSF; Vermeerbergen, 1996 for VGT).

In the studies discussed so far, researchers have often identified the movement and/or handshape as the basis of the classifier construction. Other parameters (including location, orientation, and non-manual components) are mostly seen as components that can complement the movement and handshape. This is in contrast with the conceptualization of classifier constructions as *‘mix’ ‘n match signs,’* a concept first introduced by Brennan (1990, p. 163):

(…) mix “n” match involves selecting the component parts and putting them together in appropriate ways in order to create particular kinds of effect.

From this point of view, there are no parameters that lie at the basis of the construction, but rather all parameters are equal to one another. The idea is more that signers create new signs in order to show a particular referent/event. In doing so, they select the parameters they need to prompt that meaning and put them together in the creation of a new sign (Brennan, 1990). From this point of view, classifier constructions are seen as an important part of the productive lexicon and are here also referred to with terms like *productive signs*, *classifier constructions* and *verb/sign constructions* (see for instance, Vermeerbergen, 1996).

#### 2.2.3. Gesture as an Integral Part of Language: (Partly) Gestural Constructions

In more recent studies on classifier constructions, the idea that these constructions are entirely symbolic, i.e., conventional, is questioned. This is parallel to an increased interest in the use of gesture by speakers (see for instance, Goodwin, 1981; McNeill, 1992; Streeck, 1993; Kendon, 2004). Although (some of the) researchers of the studies mentioned above might have been aware of this evolution, it seems that most of them were initially not eager to adopt a gestural analysis of classifier constructions. One of the arguments was that classifier constructions are by far more complex than gestures and thus these researchers maintained a morphemic conceptualization. The first researchers that adopted a (partly) gestural analysis of classifier constructions were Cogill-Koez (2000 for Auslan), who returns to DeMatteo’s (1977) notion of visual imagery and argues that classifier constructions are in fact entirely gestural constructions and Liddell (2003 for ASL) who proposes that classifier constructions, or rather depicting signs, are hybrids of symbolic, i.e., conventional, and gradient, i.e., gestural, properties. We will focus on Liddell’s work here, because his conceptualization is – in particular within the group of cognitive and functional signed language linguists – the most widespread and adopted conceptualization today.

Liddell (2003), who takes Mental Space Theory as a starting point (Fauconnier and Turner, 1996; Fauconnier, 1997), suggests that classifier constructions are partly lexical verbs that both encode linguistic meaning and depict meaning. He proposes that signers bring referents, i.e., conceptual entities, into Real Space, i.e., the signer’s immediate environment. When using classifier constructions, signers map the referent onto their own hands. Liddell (2003) argues that the handshapes are the more conventional part of the construction, because they can only refer to specific types of entities. The location and/or movement are then seen as the more gradient, i.e., gestural properties of the construction (see however, Ferrara, 2012). Liddell identifies three types of partly lexical classifier constructions: constructions that depict entities at a location, constructions that depict movement and constructions that depict the extent and shape of a referent. In more recent studies, constructions that depict the handling of an object are also added to the list (e.g., Johnston and Schembri, 2007; Ferrara, 2012 for Auslan).

In this section, we have shed light on the most important changes in the thinking about classifier constructions in the last (approximately) 50 years. We have shown that, although initially the iconic character of these constructions was emphasized, soon more formalist, morphemic approaches became more dominant. As a consequence, the importance of iconicity at this level of signed language discourse was downplayed or even ignored. Researchers adopting more formal approaches rather emphasized the comparability of classifier constructions with classificatory verb systems in some spoken languages. Only toward the end of the 1990s, researchers (re)discovered their iconic properties and also questioned the status of the handshapes as morphemes. Finally, as a result of the increased interest in gesture in the study of language, the “linguistic-only” approaches have been replaced with reconceptualizations of these constructions as partly lexical constructions that exhibit both symbolic and gradient properties (but see for instance, Garcia and Sallandre, 2020 for a semiologic approach, Lepic and Occhino, 2018 for a construction morphology approach). This conceptualization of classifier constructions, which is the most wide-spread and adopted approach to date, especially amongst researchers working within Cognitive Linguistics, will also be adopted in this article.

## 3. Role Shifting = Constructed Action?

Part one of this article has shed light onto the different conceptualizations of classifier constructions and role shifting in the literature, thereby also referring to concomitant terminology shifts. In this part of the paper, we further explore the relation between “role shifting” and “role playing” on the one hand, and “constructed action” on the other hand.

In recent literature, we regularly find statements such as the following:

– “CA, or enactment, also known as role-shift” (Cormier et al., 2013, p. 370).
– “A last phenomenon that needs introduction is role shift, or constructed action (CA)” (Boers-Visker, 2020, p. 50).
– “Enactment, also known as constructed action (CA) or role shift; a non-linguistic demonstration of entities and actions; a surrogate real space blend” (Ferrara and Johnston, 2012).

The question we ask here is whether this indeed merely is a matter of modernizing terminology or whether it is possible that these different notions are used to capture (slightly) different phenomena in signed language discourse.

That the notion of *role taking* would be outdated and best replaced by *constructed action* was also suggested in the early stages (around 2013) of the annotation of the Corpus Flemish Sign Language^5^. At a certain point, the team of annotators wondered if it would not be better to create a tier “CA” to replace the tier “role taking.” This question was prompted by the adaptation of Johnston’s (2011/2013) annotation guidelines for the Auslan corpus, where a tier “CA” is used for the identification of periods of time in which the signer is engaged in constructed action or constructed dialogue. Of course, the proposal to replace the role-taking tier with a CA tier raises the question of whether these two phenomena are truly identical.

As becomes clear from section “Role Playing” of this article, very different definitions exist for these two terms. Given the Flemish perspective of this article, we take the way the phenomena are usually understood in Flanders as a starting point here. The term *rolnemen* (*role taking*) was introduced in Flanders by Van Herreweghe (1995) and Vermeerbergen (1996, 1997). Van Herreweghe (1995, p. 113) uses *rolnemen* (as a translation for *role-taking*) to refer to the ways in which a signer reproduces a previous conversation with someone or reproduces what someone has said. She briefly describes four such “ways”: “role-taking by means of body shift,” “role-taking through facial expressions and body posture,” “role-taking through indexing,” and “role-taking through eye-gaze.”

Vermeerbergen (1996), who provides the first large-scale corpus study of Flemish Sign Language, starts from Engberg-Pedersen’s (1993) three-way division (see section “Role Playing”) and suggests to distinguish between *rolnemen* (*role taking*) and *referentiewissel* (*shifted reference*). *Shifted reference* here refers to the organization of spatial grammatical mechanisms as if the referent’s location were identical to the signer’s actual location while role taking involves “taking over” the role of a referent by means of non-manual behavior. Furthermore, Vermeerbergen (1996, p. 139–140) distinguishes between role taking marked by a body lean in the direction of a previously established locus for the referent (*formal role taking*), and the use of non-manual articulators to adopt a role (*expressive role taking*). She also identifies various degrees of role taking.

**FIGURE 1:**
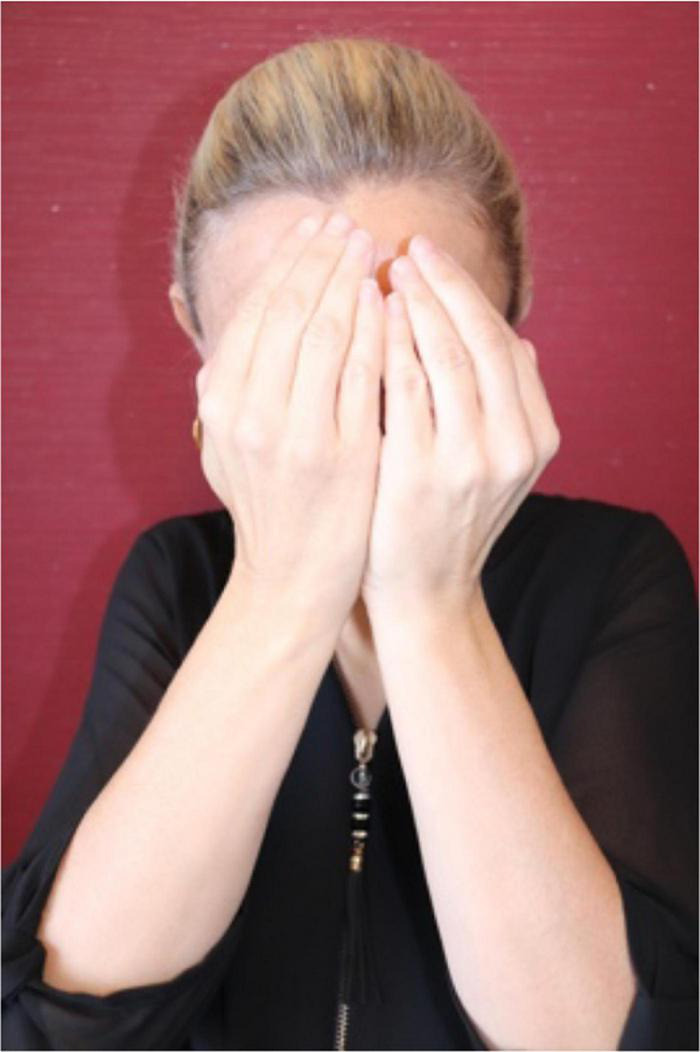
G: COVER-EYES.

**FIGURE 2:**
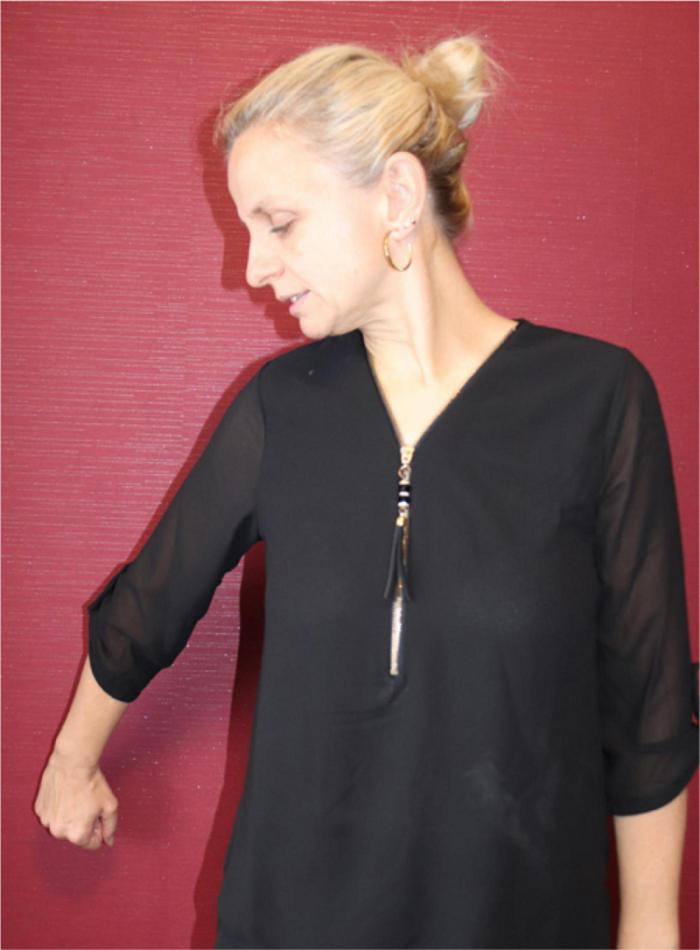
G: TAKE-BY-THE-HAND.

**FIGURE 3:**
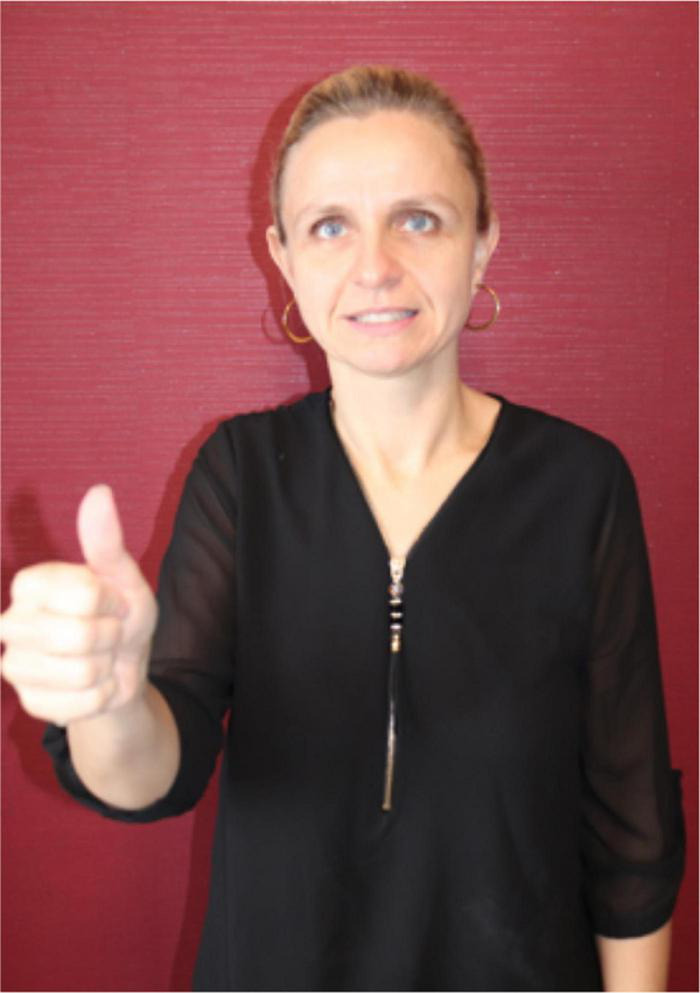
G: PUSH-BUTTON.

In light of her Ph.D. project, Vermeerbergen (1996) also identified the types of gestures that are known as *iconic gestures* (McNeill, 1992 on co-speech gesture) and *constructed action* (Metzger, 1995; Liddell and Metzger, 1998 for ASL). Some examples are: G: COVER-EYES (Figure 1), G: TAKE-BY-THE-HAND (Figure 2), and G: PUSH-BUTTON (Figure 3).

In some cases, these gestures were analyzed as what is now called *depicting handling signs*, i.e., as classifier constructions involving handling classifiers handshapes. When such an analysis was not appropriate, and especially when the gestures were to some extent similar to co-speech gestures, the construction was indeed considered gestural. Within the framework of the Ph.D. research, these were not further analyzed because it was not yet customary to pay too much attention to gesture in (signed) discourse at that time (i.e., beginning of the 1990s). Liddell and Metzger (1998, p. 694) write:

By the time ASL was demonstrated to be a real human language, virtually all the gesturing done by the hands was considered linguistic and much of the non-manual aspects of the signing were also considered linguistic. Recent analyses of head and body tilts and rotations have also viewed these behaviors as the realization of grammatical elements.

**FIGURE 4:**
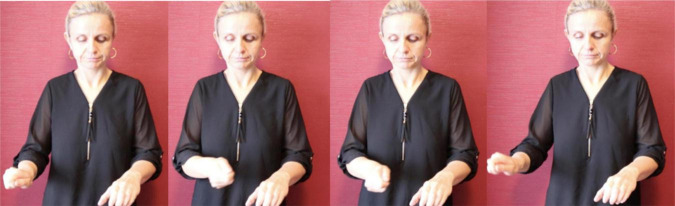
G: BRICK-A-WALL.

At the start of the annotation of the Corpus Flemish Sign Language manual iconic gestures were annotated in the way proposed in the annotation guidelines of Johnston (2011/2013, p. 34): the capital letter G, followed by a colon and the meaning of the gesture(s). During discussions amongst the annotators, (at least some of) these gestures were referred to by the term *constructed action*, but exactly how the term was interpreted was not entirely clear. At one point it was suggested to make a distinction between CA at the discourse level, where the signer may imitate the expression and/or posture of a certain referent to indicate role, and CA at a lexical level, where gestures and/or bodily behaviors are used instead of signing, e.g., to enact a certain action or posture of a referent and functioning as a predicate, e.g., G: wink [cf., Johnston and Schembri (2007, p. 258–260) on the “carpentry class narrative”] or G: BRICK-A-WALL (Figure 4) where the instances of CA might be replaced by lexical or depicting signs. The suggestion further involved calling the first form of CA *role taking*, and the second form simply *gesture* or *enacting gesture* (Vermeerbergen, 2014).

The multitude of definitions and descriptions, with sometimes confusing information as to how exactly the phenomena are understood and analyzed do not make it easier to determine whether role taking and constructed action/enactment are the same or (slightly) different. For example, even within one and the same publication, certain changes in non-manual signals are sometimes called *role shifting* while also being analyzed as “accompanying role-shifting,” or “marking role-shifting” (cf., section “Role Playing”). Below, we present our current approach, bearing in mind that further work and data analysis may reinform our understanding.

First, we propose to distinguish constructed action from constructed dialogue. Then there is the question whether role taking should be distinguished from CA. Central to role taking is, of course, the notion of “role.” In older approaches, including discussions of role taking in the literature on Flemish Sign Language from the 1990s, it is argued that body shifts can be used as signaling role (or that body shifts can be interpreted as role shifts), especially when signing about a conversation between two characters. Such episodes/stretches of body shift do not necessarily also involve the use of gestures that demonstrate the actions of the character(s) involved and/or the (re)construction of the facial expression or body position of one or more of the character(s) involved. In other words, in some instances “role shift” is marked by a slight change of the position of the body only, without any form of “enactment.” At this moment, i.e., in light of the current state of documentation of VGT, it does not seem appropriate to us to exclude the option that (signaling) role implies only body shift (possibly in combination with shifted reference) and not enactment.

Of course, role taking often involves the signer’s use of one or more articulators to “show” (“depict”) the actions, thoughts or feelings of a referent, but there are also instances where signers use a gesture/gestures to express a certain action but not necessarily a certain action *of a particular character.* When reviewing the definitions of CA in the literature (cf., “Role Playing”), it became clear that many authors include the idea that the signer is adopting a role. A definition of CA as *“the gestures that imitate the actions of someone other than the signer at the time of signing”* (Johnston and Schembri, 2007, p. 273), for instance, implies that certain gestures can only be interpreted as either actions of the signer at the time of signing or as “belonging” to a character.

We want to suggest that a third interpretation is possible, and that the signer may simply represent a certain action, without assuming a “role” (other than the narrator role). In other words, signers can also use mimetic/iconic gestures as an alternative to an established verb sign or partly lexical classifier construction. This is for example the case in descriptions of recipes or other forms of instructions where signers describe a series of actions to be performed, some expressed by fully-lexical signs, some by partly lexical classifier constructions and some by constructed action or other types of gestures.

A similar three-way split seems possible for CA in combination with speech. Liddell (2003, p. 158) discusses an example [example (11)] where the spoken words “Frank was looking for his keys” are uttered while pressing the palms against shirt pockets, then pants pockets. He writes that “the temporal coordination of the verbal description and the constructed action invites the addressee to interpret the pressing movements of the hands as searching movements. As a result, the message expressed in (11) is much more explicit than provided by the words alone” (Liddell, 2003, p. 158). Liddell continues by stating that in order to understand this example, one must see the speaker’s action as Frank’s action. “The mental space property ‘Frank’ has been projected onto the current speaker” (Liddell, 2003, p. 158). We do not mean to claim that this analysis is incorrect but rather that another interpretation is possible. Namely Frank’s action is here represented by means of speech and co-speech gesture, without there necessarily being a real-space blend. In other words: the speaker might be using the gesture in order to depict the general action of searching for the keys, without necessarily adopting the role of Frank.

To summarize, we propose to approach role-taking and constructed action as different phenomena, although often combined. Cormier et al. (2015, p. 195) make a *prima facie* similar suggestion, but they talk about the distinction between constructed action (CA) on the one hand and role shift on the other, and role shift is interpreted as a shift between different roles:

This may be a shift between a period of narration (narrator role) and a period of non- narrator role (character role) expressed via CA, or between two character roles expressed by CA and determined by the CA articulators.

The importance attributed to “role” or to identifying (character) role in recent studies of CA/enactment may be related to the data that are being analyzed. Very often these concern retellings of picture stories [e.g., *Frog, where are you* (Mayer, 1969)], cartoons (Garfield) or clips from animated movies (e.g., Wallace and Gromit) featuring a limited number of characters actively performing actions. As a consequence, these characters also have a central role in the retellings and it is often (more or less) straightforward to identify the referents that are depicted through, for instance, constructed action. However, in other types of discourse and in particular in more spontaneous conversations, the notions of role and characters might be less prominent and the idea that signers may construct action without necessarily assuming a character role might become apparent more easily.

Finally, although CA is often used to depict actions, postures, attitudes, …, signers may also use CA as an alternative for lexical or partly lexical signs with the intention to “simply say” or describe, rather than depict. Imagine a speaker saying “And then he contacted me,” while simultaneously imitating holding a phone. Here the gesture adds meaning that is not provided by the spoken message. Especially when there is no simultaneous *shifted attribution of expressive elements*, i.e., no other non-manual articulators that depict some aspect of the action, one might wonder whether the signer aimed to accurately show the action of “calling” or whether he/she rather wants to describe the action, thereby using both speech and co-speech gesture. In other words, the depictive potential of the gesture might be backgrounded. In the next section, we continue on this line and explore the relation between the methods of describing (telling), depicting (showing), and indicating (pointing and placing) on the one hand, and the different sign types in the lexico-grammar of signed languages on the other hand.

## 4. Depicting – Form or Function?

The previous section has illustrated that terminology shifts can come with certain risks. Whereas some authors have used role shifting and constructed action interchangeably, we have proposed that these might be rather two different functions. In this section, we continue on this line by focusing on the conceptualization of partly lexical classifier constructions as “depicting signs” (cf., Liddell, 2003 for ASL) and on how these constructions relate to the methods of describing (telling), indicating (pointing and placing), and depicting (showing). We first provide a brief overview of existing ideas about classifier constructions and constructed action and the relation between those two phenomena and the methods of describing, indicating and depicting. Subsequently, we raise some questions regarding the conceptualization of classifier constructions as depicting signs and their relation with the method of depicting and explore how other sign types (fully-lexical signs, indicating verbs, and pointing signs) can also be exploited to depict meaning. In doing so, we illustrate that some of the current conceptualizations of the different sign types and the terms that are used to refer to these phenomena do not accurately capture the different ways that signers use them in actual language use. Building on insights from, for instance, Ferrara and Halvorsen (2017), Capirci (2018), Ferrara and Hodge (2018), Puupponen (2019) and Beukeleers (2020), we therefore promote the idea that (1) signs in the lexico-grammar of signed languages are best considered as hybrids of descriptive, depictive and indexical properties and (2) that depicting is a semiotic diverse practice and thus that signers can draw on various types of semiotic signs in the construal of depictions.

### 4.1. Some Early Ideas About Depiction

#### 4.1.1 Classifier Constructions as Depicting Signs

As highlighted in part 1 of this article, Liddell was the first author to reconceptualize classifier constructions as depicting signs, which differ from fully-lexical signs because “in addition to their encoded meanings, these verbs also depict certain aspects of their meanings” (Liddell, 2003, p. 261). In this way, Liddell suggests that these signs are hybrids of descriptive and depictive properties.

#### 4.1.2. Depicting Blends and Surrogate Blends

A second important idea in Liddell’s (2003) work is that signers can use partly lexical classifier constructions and/or constructed action in order to create topographical real space blends. On the one hand, signers can create depicting blends, i.e., they can create small-scaled depictions of the event space they refer to in the sign space in front of them. On the other hand, signers can create a life-sized depiction in which they use their own body to depict a referent’s actions, thoughts and/or feelings. In this way, signers create a surrogate blend in which they are – in contrast to when they are creating depicting blends – no longer the narrator, but they rather physically become the referent. As such, Liddell (2003) associates the creation of depicting blends with the use of partly lexical classifier constructions and surrogate blends with the use of constructed action, also known as enactment (Metzger, 1995; Liddell and Metzger, 1998; Cormier et al., 2015; see sections “Role Playing” and “Role Shifting = Constructed Action?”).

#### 4.1.3. Transfers

**FIGURE 5:**
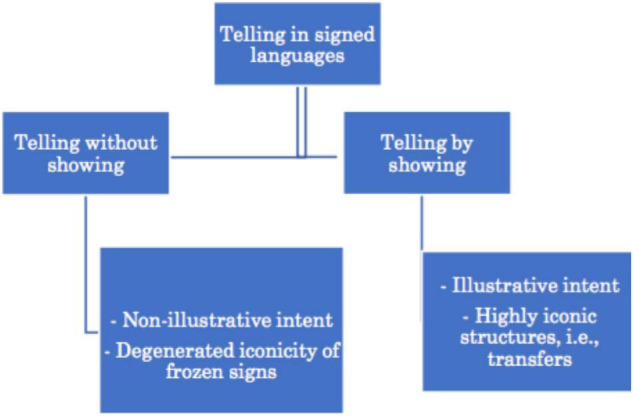
Visualization of the relation between the different modes of communication and different sign types based on Sallandre (2003).

Similar ideas with regard to the notion of depiction can be found in studies that adopt a semiological perspective to signed discourse (e.g., Cuxac, 1996, 2000; Sallandre, 2003; Cuxac and Sallandre, 2007; Sallandre, 2007; Garcia and Sallandre, 2019 for LSF). Recall from section “Role Shifting = Constructed Action?” that researchers working within this theoretical framework take the signer’s intent as a starting point. They argue that signers can choose between different modes of communication when reconstructing experiences through language: telling without showing and telling by showing. With the former, also referred to as the *non-illustrative intent*, signers mainly draw on fully-lexical signs (which they refer to as *standard signs*). When telling by showing, however, signers mainly want to show what a particular referent looks like and therefore rather use different types of *transferts* (transfers), i.e., highly iconic structures through which the signer depicts the referent in the sign space (Cuxac, 1996, 2000; Cuxac and Sallandre, 2007). Figure 5, which is based on the illustration of Sallandre (2003), visualizes the relation between the different modes of representation and the different sign types.

Besides the personal transfer discussed in section “Role Playing,” Cuxac (2000) also distinguishes between transfers of size and shape and situational transfers. To a certain degree, these types of transfers are comparable to the notion of depicting signs of movement, location, size, and/or shape (Liddell, 2003). Personal transfers, on the other hand, are – to a certain extent – comparable to the concepts of, for instance, bodily enactment (Ferrara and Johnston, 2014 for Auslan) and constructed action (Cormier et al., 2015 for BSL) (see section “Role Playing”).

### 4.2. What About Other Sign Types?

#### 4.2.1. Topographical Use of Signs Space

Although Cuxac (1996, 2000) and Liddell (2003) differ fundamentally in the way they conceive of language, especially with regard to the role of gesture therein, their conceptualizations of the construal of depictions share some interesting similarities. Both researchers mainly associate the method of depicting with the use of classifier constructions and bodily enactment. Both Cuxac and Liddell have had a great impact on the study of depictiopn wihtin the field of signed language linguistics. For instance, when reviewing empirical studies on the construal of depictions, it becomes apparent that most researchers have limited their empirical analyses to the use of partly lexical classifier constructions and/or bodily enactment (e.g., Mulrooney, 2006 for ASL; Perniss, 2007 for DGS; Engberg-Pedersen, 2015 for DTS).

This is striking because there is a large body of research that highlights the topographical use of sign space with other sign types. In other words: researchers working on different signed languages have shown that signers often incorporate topographical information about the referents in the way they structure the sign space with – for instance – pointing signs and indicating verbs, i.e., verbs that are meaningfully directed toward entities, directions, and/or places (Liddell, 2000, 2003; see also Bos, 1990 for NGT; Johnston, 1991, 1996, 2019; De Beuzeville et al., 2009 for Auslan; Engberg-Pedersen, 1993 for DTS; Vermeerbergen, 1996, 2006; Beukeleers and Vermeerbergen, 2017 for VGT; Cormier et al., 2015; Fenlon et al., 2018 for BSL). When modifying the indicating VGT-verb GO-TO, for instance, signers can use the sign space topographically in order to iconically represent the referents’ location(s) and movement(s) in the scene under discussion. In this way, they can thus depict the location and/or movement of the referent, i.e., they can show their interlocutor what the event looked like. This supports the idea that signers can also draw on other sign types when they want to depict meaning, i.e., when they want to show what (some aspect of) the referent looks like (see also Vermeerbergen, 2006, 2013, 2016; Ferrara and Halvorsen, 2017; Ferrara and Hodge, 2018; Beukeleers, 2020 for VGT).

#### 4.2.2. (De-)lexicalization Processes

The idea that signers can depict meaning with more conventionalized forms has also been described in terms of (de-)lexicalization processes. It has been argued that classifier constructions and stretches of non-lexical bodily enactment over time can develop into fully-lexical signs (or standard signs), i.e., through repeated use these constructions can acquire an identifiable citation form that prompts the same meaning or set of meanings across different contexts of use (e.g., Engberg-Pedersen, 1993 for DTS; Vermeerbergen, 1996, 2006, 2016 for VGT; Cuxac, 1999, 2000 for LSF; Johnston and Schembri, 1999, 2007, Vermeerbergen, 2016; Johnston and Ferrara, 2012 for Auslan; Shaffer and Janzen, 2002; Janzen, 2012 for ASL). These conventionalized signs, however, do not completely lose their iconic properties and because of that signers can always de-lexicalize them. In other words, they can always re-activate the latent iconicity of these conventionalized signs within a particular context of use in order to show their interlocutors what the referent(s) look(s) like.

The existence of lexicalization and de-lexicalization processes might indeed explain the origins of some of the highly iconic fully-lexical signs and the modification of these conventionalized signs for the purpose of depicting in signed discourse. However, it is not always easy – as an analyst – to determine the degree of conventionalization of a particular token, especially when no extensive lexical databases are available. Moreover, very often there is no historical evidence for all these signs, i.e., there is no empirical data that supports the idea that a particular token over time has developed into a fully-lexical sign (see also Cormier et al., 2012 for BSL, Ferrara and Halvorsen, 2017 for NTS, Beukeleers, 2020 for VGT). So even though (de-)lexicalization processes can often explain how signers use particular signs for the purpose of depiction, it remains difficult to apply this to the actual annotation of signed language data. In this article, we therefore provide an alternative account, i.e., we adopt a functional, semiotic point of view to the study of signed discourse (e.g., Ferrara and Halvorsen, 2017 for NTS, Capirci, 2018; Ferrara and Hodge, 2018; Slonimska et al., 2021 for LIS; Puupponen, 2019 for FinSL; Beukeleers, 2020 for VGT; see also Clark, 1996, 2003, 2016, 2019; Enfield, 2009; Dingemanse, 2011, 2013, 2014, 2017 for spoken languages). Building on insights from these studies, we suggest that depicting is a method of communication for which signers can draw on various types of semiotic signs. In the following sections, we will elaborate on this recent development by (1) presenting the functional, semiotic framework that has been adopted to the study of depiction and (2) elaborating on the resources that signers can draw on in the construal of depictions.

### 4.3. Toward a Semiotic Diversity of Depiction: A Functional, Semiotic Account to (Signed) Language

#### 4.3.1. Language as Describing, Indicating and Depicting

Linguists working on both spoken and signed languages have adopted Peirce’s (1894, 1955) semiotics to the study of language use. In doing so, they study language as a form of social action in which people rely on a range of different semiotic resources that differ in degree of conventionalization (e.g., Clark, 1996, 2003, 2016; Enfield, 2009; Dingemanse, 2015, 2017; Hsu, 2021 for spoken languages; Ferrara, 2012; Hodge, 2013; Johnston, 2013; Ferrara and Halvorsen, 2017; Ferrara and Hodge, 2018; Puupponen, 2019; Beukeleers, 2020 for signed languages). They thereby consider the different bodily articulators that are at play, such as hands, head and body movements, and eye gaze and analyze how they are brought together in the creation of larger communicative moves, i.e., composite utterances (e.g., Clark, 1996; Enfield, 2009; Ferrara, 2012; Hodge, 2013; Janzen, 2017; Ferrara and Hodge, 2018; Johnston, 2019). This section is limited to a discussion of the methods of describing, indicating and depicting. For recent discussions of Peirce’s work itself, we refer the reader to Ferrara and Hodge (2018); Puupponen (2019) and Beukeleers (2020).

Based on the different types of semiotic signs introduced by Peirce (1894, 1955)^6^, it has been suggested that people signal meaning through the methods of describing, indicating and/or depicting (e.g., Clark, 1996; Enfield, 2009; Ferrara and Hodge, 2018). When describing meaning, people represent the object categorically, i.e., they communicate by telling. This method has often been described in terms of the use of symbols, such as conventionalized words and fully-lexical signs because they have been associated with their referent by rule and the interlocutor thus needs to interpret the P-sign by decoding its meaning.

The method of indicating entails that people are locating an utterance in space and time by creating an index for the object they refer to Clark (1996, 2003), Enfield (2009), Ferrara and Hodge (2018). The speaker/signer thus uses the P-sign to point toward the object it stands for and, in doing so, he/she anchors the communicative utterance to the real world. Indicating is mainly associated with the use of indices, i.e., partly lexical forms that “glue things together” (Enfield, 2009: 13). People can indicate a referent by means of, for instance, pointing signs and pointing gestures (Clark, 2003; Liddell, 2003; Enfield, 2009; Johnston, 2013), but also with their lips (Clark, 2003; Enfield, 2009), eye gaze and head and body movements (Clark, 2003; Enfield, 2009; Puupponen, 2019 for FinSL).

Finally, the method of depicting allows people to show their interlocutor what the object looks, sounds or feels like (Clark, 1996, 2016, 2019; Enfield, 2009; Dingemanse, 2011, 2014, 2015, 2017; Ferrara and Hodge, 2018; Hsu, 2021). Speakers and signers than create a physical analog of the object in the here-and-now. They thereby combine different elements that stand in for the referents in the depicted scene. Depictions are not interpreted through decoding processes, but rather through the process of imagining: addressees aim to imagine what the object sounds, feels or looks like (Clark, 1996, 2016, 2019; Enfield, 2009; Dingemanse, 2015; Ferrara and Hodge, 2018). The method of depicting is therefore mainly captured in terms of the use of iconic P-signs, such as manual iconic gestures (McNeill, 1992; Kendon, 2004), classifier constructions (Liddell, 2003 for ASL; Johnston and Schembri, 2007, 2010; Ferrara, 2012 for Auslan), and gestural enactment (e.g., Metzger, 1995; Liddell, 2003 for ASL; Cormier et al., 2015 for BSL; McNeill, 1992; Clark, 1996, 2016; Kendon, 2004; Ferrara and Johnston, 2014; Stukenbrock, 2014; Stec et al., 2016 for spoken languages).

As describing, indicating and depicting are methods of communication, they can co-occur within a P-sign. Some P-signs integrate, for instance, the methods of describing and depicting [see Liddell (2003) on partly lexical classifier constructions, Dingemanse (2011, 2014, 2017) and Clark (2019) on ideophones]. Moreover, a large body of research has shown that signers tend to combine different signs of different types in larger communicative moves, i.e., they tend to create larger composite utterances (e.g., Clark, 1996; Enfield, 2009; Ferrara, 2012; Hodge, 2013; Ferrara and Hodge, 2018; see also Vermeerbergen, 1996, 2006 for VGT; Johnston et al., 2007 for Auslan, VGT and ISL, Jantunen, 2008, 2017 for FinSL on the integration of different types of signs in signed utterances). Thus, describing, indicating and depicting are fundamentally different methods of communication, but people tend to combine them in the creation of composite P-signs. In this regard, it should be noted that the co-occurrence of the different methods in various types of P-signs does make it difficult to isolate them and distinguish between them in actual language use.

### 4.4. Exploring Depicting as a Method of Communication

As highlighted in section “What About Other Sign Types?,” depictions are best considered as iconic renditions of the object they stand for, which allow the interlocutor to imagine what the object looks, sounds, feels like (e.g., Clark, 1996, 2016, 2019; Enfield, 2009; Dingemanse, 2015; Ferrara and Hodge, 2018). A review of the Signed Language Linguistics on this topic, has revealed that the construal of depictions has mainly been associated with and analyzed by annotating less conventionalized, less conventional, highly iconic structures, i.e., classifier constructions and constructed action. Indeed, even researchers who take the signer’s intent as a starting point analyze depiction by singling out these particular sign types. In this section, we move away from this approach by taking the semiotic framework as a starting point. In doing so, we revise existing analyses of the different sign types and argue that depicting is a property of different sign types (section “Depicting as a Property of Different Sign Types”). In other words, we argue that signers have a range of different semiotic resources at their disposal in order to depict meaning. Moreover, we also question the idea that the main function of a classifier construction, also known as a depicting sign, is always depiction. In section “Describing With Partly-Lexical Classifier Constructions,” we rather suggest that these constructions too are best analyzed within their particular context of use because signers can use them to describe, indicate and depict meaning in varying degrees.

#### 4.4.1. Depicting as a Property of Different Sign Types

In this section, we illustrate that signers can use different types of signs in the construal of depictions. While reviewing the different sign types, we show that describing, indicating and depicting are properties of different sign types, regardless of their degree of conventionalization.

##### 4.4.1.1. Fully-Lexical Signs

Ferrara and Halvorsen (2017 for NTS) were the first sign language linguists that have adopted the functional, semiotic framework in the analysis of depictions with conventionalized form-meaning pairings. Building on insights from Dingemanse’s (2015; 2017) analysis of ideophones, they propose that iconic fully-lexical signs integrate both descriptive and depictive properties which can be made manifest to varying degrees. Below, we will illustrate their analysis with two different uses of the fully-lexical sign TREE in VGT. This first example is taken from a retelling of the narrative “Frog, where are you?” (Mayer, 1969) in the Corpus VGT (Van Herreweghe et al., 2015). The signer is elaborating on the scene where the boy and the dog find a tree log in the woods.


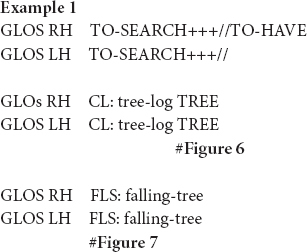


**FIGURE 6:**
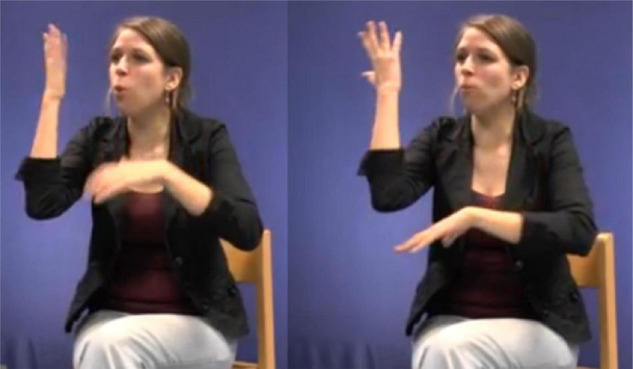
The fully-lexical sign TREE in its citation form, as produced in example 1. Figure reproduced with permission from Corpus VGT (Van Herreweghe et al., 2015).

**FIGURE 7:**
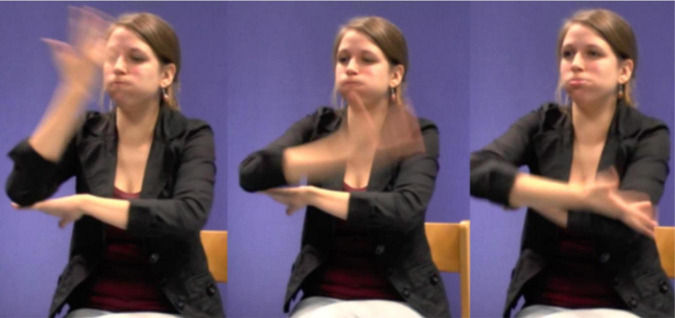
The modified fully-lexical sign FLS: tree-falls-down, as produced in example 1. Figure reproduced with permission from Corpus VGT (Van Herreweghe et al., 2015).

According to Ferrara and Halvorsen (2017 for NTS), iconic fully-lexical signs, like the sign TREE, are composites of depictive and descriptive properties. The descriptive properties can be traced back to the fact that they are conventionalized form-meaning pairings. The depictive properties, on the other hand, lie in the iconic nature of these signs. Within a particular context of use, signers can foreground one of both functions. In the first instance of the sign TREE (Figure 6), for instance, the signer uses the sign in its citation form, i.e., she produces it on a neutral location in the sign space and with a hand-internal movement only. This manual sign is accompanied by the conventional mouthing “boom,” which describes the meaning tree. In the second instance, however, the signer produces the sign with a downward movement that depicts how the tree falls down. Moreover, the signer “adds sound” to the depiction by simultaneously blowing air out of her mouth and bulging her cheeks. From a functional, semiotic point of view, it can then be argued that the signer foregrounds a descriptive reading when using the citation form (Figure 6), i.e., she mainly uses the sign in order to tell about the referent. When modifying the token in a way that it depicts the falling movement, however, she foregrounds a depictive reading (Figure 7).

Building on these insights, we suggest that iconic fully-lexical signs that can be placed meaningfully in the sign space are also best understood as composites of descriptive, depictive and indexical properties that can be made manifest to varying degrees (cf., Beukeleers, 2020 for VGT). Consider the following example:


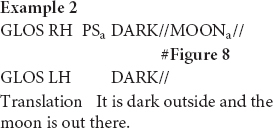


**FIGURE 8:**
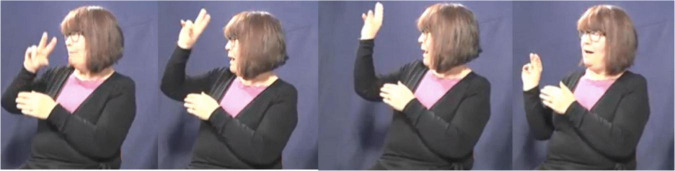
Fully-lexical sign MOON which is modified in order to indicate and depict its location, as produced in example 2. Figure reproduced with permission from Corpus VGT (Van Herreweghe et al., 2015).

In this excerpt, the signer is setting up the scene. She mentions it is dark outside and subsequently uses the fully-lexical sign MOON in order to say that the moon is already out there. As she thus uses a conventionalized form-meaning pairing and combines the manual production of the sign with the conventional word “moon” in Dutch (maan), the signer accurately describes the meaning of the referent she is introducing. However, the sign MOON is also an iconic sign that also depicts its shape. Just like the fully-lexical sign TREE, the sign MOON is thus best conceptualized as a hybrid of depictive and descriptive properties. Within this particular context of use, the signer modifies the sign and places it higher up in the sign space (see Figure 8). In this way, the signer does not only depict the referent’s shape, but also indicates its location. Hence, the sign MOON is therefore best conceptualized as a composite of descriptive, depictive and indexical properties that can be made manifest to varying degrees. In this setting the signer is setting up the space and in doing so, she is showing what the boy’s bedroom looks like. She places MOON higher up than its location in the citation form, and on the right side of the sign space. This locus reflects the position of the moon from the boy’s vantage point in the original narrative. Thus, by localizing the moon, she does not only indicate this referents’ position, but also depicts its location in the scene. The depictive function of this token becomes also apparent when considering the sign lengthening of MOON. Within this usage event, we therefore suggest that the signer foregrounds the depictive and indexical properties of MOON. This is also supported by the fact that the signer gazes in the direction of the projected referent.

##### 4.4.1.2. Indicating Verbs and Pointing Signs

Continuing on this line, we also aim to explore other sign types that signers can draw on when building a depiction. Based on research on the motivated, often topographical use of sign space in various signed languages, we argue that signers can also use indicating verbs and/or pointing signs to depict movements and/or locations. In doing so, we counter the idea that these sign types are merely hybrids of descriptive and indicative properties, an analysis first proposed by Liddell (2000, 2003). Rather, indicating verbs, just like iconic fully-lexical signs, consist of depictive, descriptive and indexical properties that can be fore- or backgrounded to varying degrees (cf., Beukeleers, 2020).

The example below illustrates how a signer can use a pointing sign in order to create a depiction.


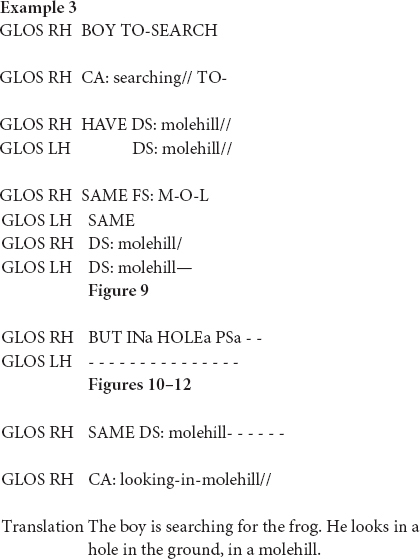


**FIGURE 9:**
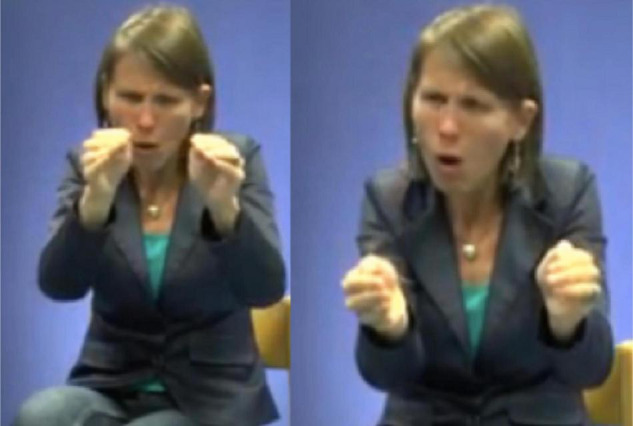
DS: molehill – classifier construction that depicts the shape of the molehill. Figure reproduced with permission from Corpus VGT (Van Herreweghe et al., 2015).

**FIGURE 10:**
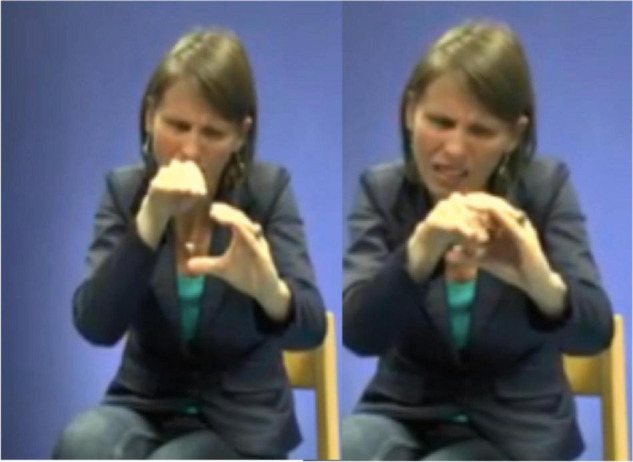
Modified fully-lexical sign IN. Figure reproduced with permission from Corpus VGT (Van Herreweghe et al., 2015).

**FIGURE 11:**
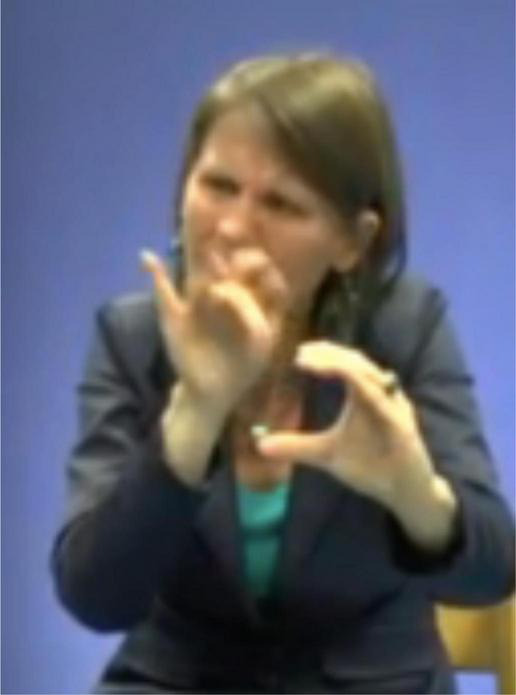
Modified fully-lexical sign HOLE. Figure reproduced with permission from Corpus VGT (Van Herreweghe et al., 2015).

**FIGURE 12:**
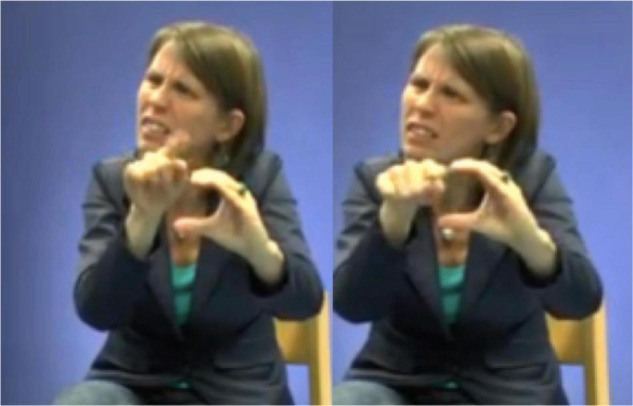
PS_*a*_ – pointing sign that traces the shape of the molehill. Figure reproduced with permission from Corpus VGT (Van Herreweghe et al., 2015).

In this excerpt, the signer introduces the molehill in the woods. She sequentially and simultaneously combines different types of semiotic signs in order to depict the scene. Toward the end of the excerpt the signer uses a classifier construction that traces the shape of the molehill (Figure 9), holds the sign and subsequently modifies the signs IN_*a*_ and HOLE_*a*_ in relation to the molehill (Figures 10, 11). Finally, she uses a pointing sign that traces the opening of the molehill (Figure 12). The signer therefore uses a more conventional index-handshape that points toward its referent. In this way, the pointing sign is simultaneously describing and indicating meaning. However, in this particular context of use the signer produces the pointing sign with a circular movement. In this way, the signer emphasizes the form of the opening of the hole in the original narrative and thus foregrounds a depictive reading.

In a similar vein, signers can use indicating verbs to describe, indicate and/or depict (some aspect of) their meaning. In the following example, the signer is reconstructing the scene where the boy is searching for the frog. In doing so, she also uses a modified token of the indicating verb TO-LOOK-AT.

**FIGURE 13:**
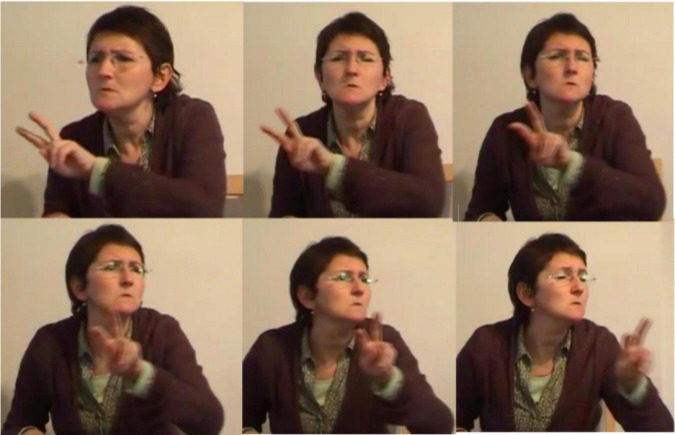
Modified indicating verb TO-LOOK-AT, as produced in example 4.

Liddell (2000, 2003) was the first linguist to analyze indicating verbs as descriptive-indexical hybrids. TO-LOOK-AT, for instance, is a verb with a recognizable citation form that prompts the same meaning across different contexts of use, i.e., it accurately describes the meaning of “looking.” Signers can, however, modify this verb in order to point toward the entity that is being looked at (e.g., Figure 13). Liddell (2000, 2003) therefore argues that these verbs do not only describe meaning, but also indicate meaning. This analysis, however, does not always capture how signers use indicating verbs in signed discourse, like in the excerpt below.


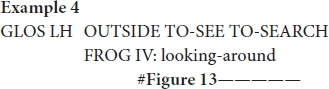


In this example, the signer is enacting how the boy is searching for the frog. During this period of enactment, the signer also produces a modified token of the indicating verb IV: looking-around (Figure 13). Within this context of use the signer thus uses a conventional token that describes the meaning of “looking.” In this excerpt, however, the signer does not use the indicating verb to point toward a particular location or entity, but she rather produces the verb with a sideward movement that shows how the boy is looking around in order to find the frog. In doing so, the signer thus accurately describes the action of the boy (i.e., looking), but also depicts the searching trajectory of the boy. The indicating verb IV: looking-around presented in Figure 13 is thus best understood as a token with descriptive, indexical and depictive properties that is modified in order to foreground a depictive, and to a lesser extent also a descriptive reading. The indicative properties are rather latent.

#### 4.4.2. Describing With Partly Lexical Classifier Constructions

In the previous section, we have shown that the construal of depictions in signed discourse cannot be captured accurately when looking at the use of partly lexical classifier constructions, i.e., depicting signs, and constructed action or enactment only. Rather, signers have a toolbox with different types of semiotic signs which they can manipulate within a particular context of use in order to foreground the descriptive, depictive and/or indexical reading. This semiotic account of depiction does then also have certain implications for the analysis of partly lexical classifier constructions. Recall from section “Classifier Constructions” that Liddell (2003) has conceptualized classifier constructions (or rather depicting signs in his terminology) as hybrids of descriptive and depictive properties. According to him, these signs thus differ from fully-lexical signs because they do not only encode linguistic meaning, but also exhibit more gradient properties that depict some aspect of their meaning. In section “Some Early Ideas About Depiction,” we have already shown that many researchers have emphasized the iconic nature of these constructions and that they have mainly been associated with the method of depicting.

From a functional, semiotic point of view, however, we may ask the question whether this conceptualization accurately captures the variety in the use of these constructions in signed language discourse. First, it is well-known that signers often place classifier constructions meaningfully in the sign space in order to reflect the position of the referent(s) in the original narrative (e.g., Liddell, 1990, 2003 for ASL; Engberg-Pedersen, 1993 for DTS; Vermeerbergen, 1996; Beukeleers and Vermeerbergen, 2017 for VGT; Johnston and Schembri, 1999, 2007 for Auslan). Within the semiotic account, classifier constructions are then not only describing and depicting, but also indicating.

Second, the question also arises whether signers indeed always mainly want to depict meaning when using these constructions (cf., Vermeerbergen, 2013, 2016; Beukeleers, 2020 for VGT). A first important consideration here is the existence of lexical gaps in, for instance, Flemish Sign Language (Van Herreweghe and Vermeerbergen, 2003; Vermeerbergen, 2013). If a signer wants to describe a referent, but there is no conventionalized form-meaning pair available, he/she will have to rely on other sign types to do so. If he/she then chooses to use a partly lexical classifier construction, does that by definition imply that he/she mainly wants to depict meaning? Or can he/she foreground the depictive properties? Moreover, if a signer does not know a fully-lexical sign for a particular concept, can’t he/she then also use a partly lexical classifier construction and foreground a descriptive reading of that token? Finally, when annotating VGT data, it becomes clear that signers use the same or similar classifier constructions in various ways. Moreover, there also seems to be intrapersonal variation in the use of classifier constructions.

Considering these critical remarks, we rather argue that partly lexical classifier constructions are composites of descriptive, depictive and indexical properties that can be fore- or backgrounded within a particular usage event. Signers can thus manipulate these constructions in order to meet their communicative aims. Example 5 below shows how a signer uses a similar partly lexical classifier construction in various ways, i.e., how the signer foregrounds different functions with the various constructions.


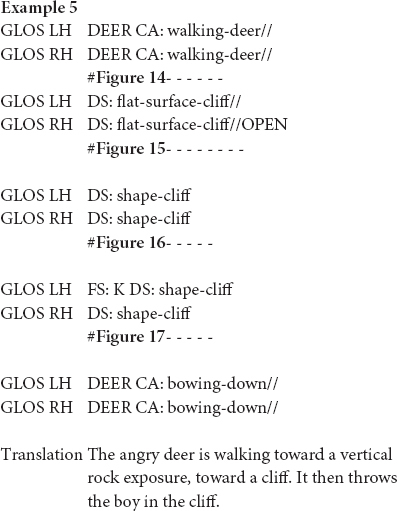


In the excerpt above, the signer is reconstructing the cliff in the story “Frog, where are you?” In doing so, she uses different partly lexical classifier constructions that trace the shape of the cliff. First, she uses a flat B-handshape to trace a flat surface, i.e., the onset of the cliff (Figure 15). She continues with the fully-lexical sign OPEN and then traces the steep slope of the cliff (Figure 16). The classifier constructions contain the more conventional B-handshape and thus describe some aspect of their meaning. Yet, as this is an iconic handshape and it is in both cases combined with a more gradient movement that traces the shape of the cliff, the classifier constructions also depict some aspect of their meaning. The fact that the signer uses two constructions that each depict a different aspect of the rock formation and that these constructions are also sequentially combined with the fully-lexical sign OPEN, might be interpreted as cues that the iconic features, i.e., the shape of the cliff, are foregrounded. In other words, the signer emphasizes what the cliff looks like and thus emphasizes a depictive reading.

**FIGURE 14:**
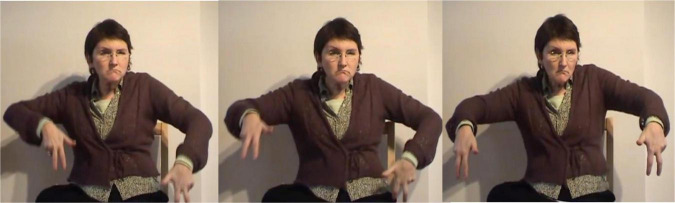
Stretch of constructed action (CA: walking-deer), as produced in example 5.

**FIGURE 15:**
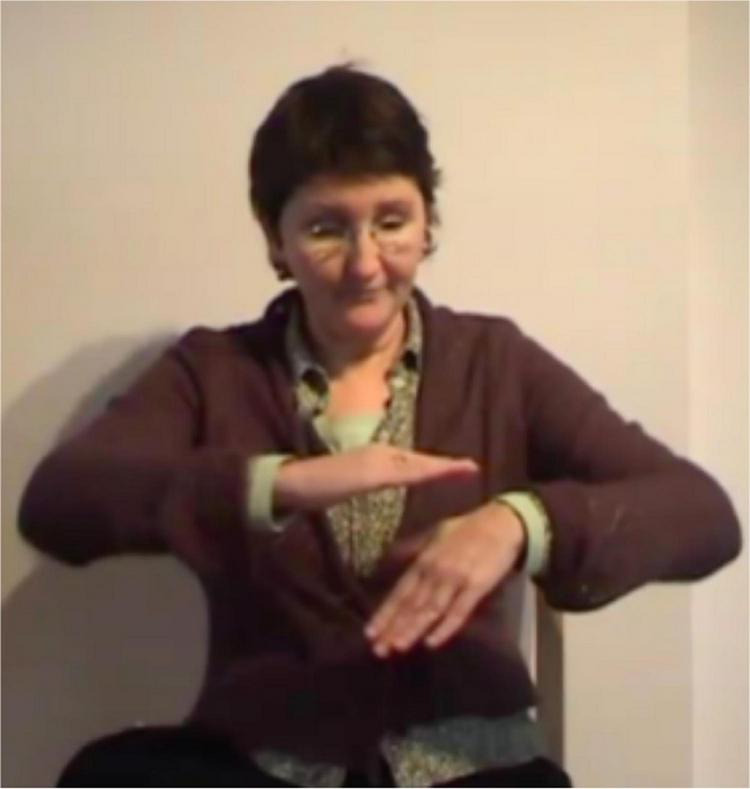
Classifier construction that traces a flat surface in the reconstrual of a cliff.

**FIGURE 16:**
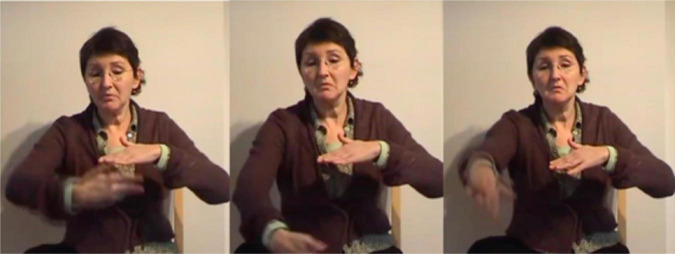
Classifier construction that traces the shape of the cliff.

**FIGURE 17:**
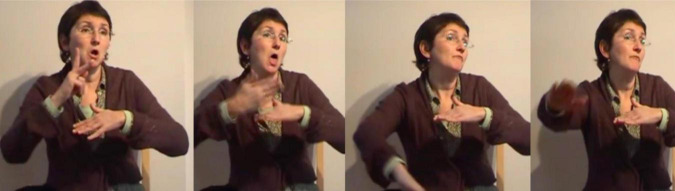
Fingerspelling “K” and repetition of classifier construction that traces the shape of the cliff.

This is slightly different for the final classifier construction, presented in Figure 17. The signer first fingerspells the letter “K” and subsequently repeats the classifier construction that traces the slope of the cliff, while simultaneously mouthing the Dutch word for cliff (klif). Just like the previous classifier constructions in this excerpt, the token is best understood as a composite, semiotic sign that integrates descriptive, depictive and indicative properties. Within this particular context of use the signer uses both conventional fingerspelling and mouthing in order to narrow down the potential meaning of the construction and thus to specify what the object exactly is. Hence, the signer accurately describes what the construction stands for and she thus foregrounds a descriptive reading.

#### 4.4.3. Summary

In sum, we have shown that the methods of describing, indicating and depicting in the sign linguistic literature have mainly been associated with the use of particular sign types, i.e., fully-lexical signs, indicating verbs + pointing signs, and partly lexical classifier constructions (i.e., depicting signs) + constructed action, respectively. Focusing on the relation between form and function, we have argued that depiction is a semiotic diverse practice (cf., Ferrara and Halvorsen, 2017; Ferrara and Hodge, 2018; Beukeleers, 2020; see also Clark, 1996, 2016, 2019; Dingemanse, 2011, 2014, 2015, 2017; Hsu, 2021 for spoken languages). In other words: we have shown that when signers want to depict meaning, they have different types of signs at their disposal, which they can manipulate in various ways in order to describe, indicate and/or depict to varying, but complementary degrees. Continuing on that line, we have also questioned the idea that the main function of a classifier construction is always depicting (cf., Vermeerbergen, 2013, 2016; Beukeleers, 2020 for VGT) and we have shown that signers can also foreground a descriptive reading of partly lexical classifier constructions, i.e., they can tell about the referent without emphasizing its iconic characteristics in a particular usage event.

In that way, this section thus also indicates that it might be misleading to name particular forms after functions. The researcher(s) introducing a term like “indicating verbs” might be aware of the fact that other types of signs can also be used to indicate meaning or that these signs do not always have to indicate. However, this nuance can easily fade away in later publications and what is mainly left is a term that does not always capture the way signers use these signs in actual signed discourse.

## 5. Conclusion

In this article, we have presented a brief overview of the study of partly lexical classifier constructions/depicting signs on the one hand, and role shifting/constructed action/enactment on the other hand. In the first part of this article, we shed light on the evolutions in the conceptualization of these phenomena and the concomitant terminology shifts. While doing so, we have shown how the symbolic, morphologic conceptualizations of role shift and classifier constructions have been reconsidered and how more recently, especially in work of researchers working from a cognitive, functional point of view, these constructions have been reconceptualized as (partly) lexical constructions. In doing so, they have promoted the role of gesture in language and rediscovered the importance of iconicity in signed discourse. Along with these theoretical evolutions, we have shed light on the terminology shifts in the literature. In more recent studies, classifier constructions are more often referred to with the term “depicting signs” and role shifting with, for instance, “constructed action” and “enactment.” Many authors seem to imply that these older and newer terms can be used interchangeably.

In part 2, we have shown that these terminology shifts can come with certain risks. We pointed out that care should be taken when replacing older terms that have often been taken from studies adopting more formal approaches with newer terms that come from Cognitive Linguistics and Gesture Studies. Do the new terms indeed refer to the same mechanisms and/or functions? And are they thus indeed comparable? Or might it be that the terms refer to different mechanisms and/or functions? We have illustrated the importance of these questions in our discussion of the relation between role shifting and constructed action. Whereas it was sometimes assumed in recent studies that these terms concern the same function, we have shown that signers can take on a character role by (1) means of body and/or eye gaze shifts only, or (2) by means of other non-manual articulators depicting the actions, thoughts, feelings or utterances of a referent, i.e., through constructed action. Signers can thus use constructed action to take a role, i.e., to depict the actions of a particular character. This latter practice is very often reported on in studies on CA and is often integrated as a feature of CA in the definitions. However, we have also highlighted that signers can construct action without actually adopting a character role. Therefore, we have argued that CA and role shifting are different, but related functions.

Finally, we have explored the relation between the functions of describing, depicting and indicating on the one hand, and prototypical forms that have often been associated with them, i.e., (1) fully-lexical signs, (2) partly lexical classifier constructions (i.e., depicting signs) and constructed action, and (3) indicating verbs and pointing signs, respectively. Focusing on the method of depicting, we have argued that signers can use different types of signs in order to depict meaning. In other words, signers can modify fully-lexical signs, indicating verbs and pointing signs in order to foreground the depictive and/or indexical properties in order to show their interlocutor what the (imaginary) event exactly looks/looked like. On top of that, we also showed that the main function of a stretch of enactment or a partly lexical classifier construction does not always have to be depicting. Signers can manipulate these tokens too in order to foreground a descriptive (and indexical) reading. We thus argue that fully-lexical signs that can be modified for movement and/or location, indicating verbs, pointing signs and partly lexical classifier constructions (i.e., depicting signs) exhibit descriptive, depictive and indexical properties that can be made manifest to varying degrees (cf., Ferrara and Halvorsen, 2017 on iconic signs in NTS, Beukeleers, 2020 on VGT). These sign types should thus be analyzed within their context of use in order to accurately capture their main function(s). With these reconceptualizations, we also highlight the importance of making a clear distinction between form and function and we emphasize that is important to be cautious when assuming a (too) exclusive relation between a certain function and particular forms.

We have written this article based on our experience with the analysis of Flemish Sign Language data. It remains an open question whether all our ideas are applicable to all other signed languages. We should keep in mind that signed languages may differ here cross-linguistically [see also Quer (2018) on quotational and non-quotational role shift in different signed languages]. However, we believe that this article provides an important contribution to the field of Signed Language Linguistics as the sign types we discussed, including constructed action and partly lexical classifier constructions, occur in other signed languages as well and it thus opens doors for cross-linguistic comparison. Important questions that arise then are, for instance: Are constructed action and role shift two different functions in all signed languages? Or are there rather signed languages for which it can be argued that they are one and the same function? Moreover, our reconceptualization of depiction also creates opportunities to analyze which resources signers actually use when building depictions and to compare this cross-linguistically.

## Author Contributions

MV and IB conceptualized the study and wrote part 1 together. MV mainly focused on the literature on role shifting and IB on the literature on classifier constructions. MV took the lead in drafting part 2 of the manuscript. IB took the lead in drafting parts 3 and 4, and conclusion. Both authors have provided feedback on the different parts of the manuscript and worked on the revision.

## Conflict of Interest

The authors declare that the research was conducted in the absence of any commercial or financial relationships that could be construed as a potential conflict of interest.

## Publisher’s Note

All claims expressed in this article are solely those of the authors and do not necessarily represent those of their affiliated organizations, or those of the publisher, the editors and the reviewers. Any product that may be evaluated in this article, or claim that may be made by its manufacturer, is not guaranteed or endorsed by the publisher.

## Footnotes

1 We adopt the view that classifier constructions are partly lexical signs (e.g., Liddell, 2003; Ferrara, 2012). However, we use the term “classifier constructions” rather than depicting signs, because part 3 of this article investigates how “depicting” can also be a characteristic of other sign types. We therefore believe that the use of “depicting signs” might be confusing.

2 See the abbreviations section above for the signed languages that the abbreviations refer too.

3 See Appendix for transcription conventions.

4 An interesting observation here is that some of these definitions also apply to “handling depicting signs,” since in the latter constructions too, the signer uses his own hands/arms to refer to actions, often the actions of someone else.

5
www.corpusvgt.be

6 Peirce (1894, 1955) conceptualizes a semiotic sign as tokens that refer to an object and are addressed to someone. P-signs, i.e., signs in Peirce’s understanding of signs, are thus in a tripartite relation between the sign (a form), the object it stands for and the interpretant (the reaction after a sign is interpreted as a P-sign). Based on the relation between the sign and the object, Peirce distinguishes between symbols, icons and indices.

## Appendix

Transcription conventions

**Table:**

WHAT	GLOS, i.e., the English word used to refer to the meaning of a VGT sign.
PS	Pointing signs.
PRO.3	Pronominal pointing sign that refers to a third person.
DS	classifier constructions (i.e., depicting sign).
FLS: leaving-bedroom	Modified fully-lexical sign that depicts movement and/or location.
IV: going-to	Modified indicating verb to depict movement and/or location.
#Figure 2	Illustration of depiction in the transcript as shown in Figure 2.
/	syntactic or prosodic break in the utterance.
//	End of the basic discourse unit.

## Abbreviations

ASL: American Sign Language
Auslan: Australian Sign Language
BSL: British Sign Language
DTS: Danish Sign Language
LSF: French Sign Language
LIS: Italian Sign Language
NTS: Norwegian Sign Language
SSL: Swedish Sign Language
VGT: Flemish Sign Language.

## References

[B115] Bendixen B. (1975). *Eye Behaviors Functioning in American. Sign Language*. San Diego: Salk Institute and University of California.

[B1] Beukeleers I. (2020). *On the Role of Eye Gaze in Flemish Sign Language: a Multifocal Eye-Tracking Study on the Phenomena of Online Turn Processing and Depicting.* Ph. D. thesis. Leuven: University of Leuven.

[B2] Beukeleers I. Vermeerbergen M. (2017). Raumnutzung in der Flämischen gebärdensprache: eine vergleichende studie zum einfluss des elizitierungsmaterials. *Das Zeichen* 107 468–477.

[B3] Boers-Visker E. (2020). *Learning to use Space. A study into the SL2 Acquisition Process of Adult Learners of Sign Language of the Netherlands.* Ph. D. thesis. Amsterdam: LOT.

[B4] Bos H. (1990). “Person and Location Marking in Sign Language of the Netherlands: Some implications of a Spatially Expressed Syntactic System,” in *Current Trends in European Sign Language Research: Proceedings of the Third European Congress on Sign Language Research*, eds Prillwitz S. Vollhaber T. (Hamburg: Signum Verlag), 231–246.

[B5] Brennan M. (1990). *Word formation in British Sign Language.* Stockholm: University of Stockholm Press.

[B6] Brennan M. (1992). “The visual world of BSL: an introduction,” in *Dictionary of British Sign Language/English*, ed. Brien D. (London: Faber and Faber), 1–133.

[B7] Capirci O. (2018). “Visible bodily action in the emergence and development of speakers’ and signers’ languaging,” in *A Paper Presented at the ISGS 8 Conference. Cape Town, South Africa, July 4–8, 2018*, (Cape Town: ISGS).

[B8] Clark H. H. (1996). *Using Language.* Cambridge: Cambridge University Press, 10.1017/CBO9780511620539

[B9] Clark H. H. (2003). “Pointing and placing,” in *Where Language, Culture and Cognition Meet*, ed. Kita S. (Mahwah, NJ: Lawrence Erlbaum Associates), 243–268.

[B10] Clark H. H. (2016). Depicting as a method of communication. *Psychol. Rev.* 123 324–347. 10.1037/rev0000026 26855255

[B11] Clark H. H. (2019). “Depicting in Communication,” in *Human Language: From Genes and Brains to Behavior*, ed. Hagoort P. (Cambridge, MA: MIT Press), 235–247.

[B12] Cogill-Koez D. (2000). Signed language classifier predicates: linguistic structures or schematic visual representation? *Sign Lang. Linguist.* 3 153–207. 10.1075/sll.3.2.03cog 33486653

[B13] Cormier K. Quinto-Pozos D. Sevcikova Z. Schembri A. (2012). Lexicalisation an de-lexicalisation in sign languages: comparing depicting constructions and viewpoint gestures. *Lang. Commun.* 32 329–348. 10.1016/j.langcom.2012.09.004 23805017 PMC3688355

[B14] Cormier K. Smith S. Sevcikova Z. (2013). Predicate Structures, gesture, and simultaneity in the representation of action in british sign language: evidence from deaf children and adults. *J. Deaf Stud. Deaf Educat.* 18:ent020. 10.1093/deafed/ent020 23670881 PMC3943391

[B15] Cormier K. Smith S. Sevcikova Z. (2015). Rethinking constructed action. *Sign Lang. Linguist.* 18 167–204. 10.1075/sll.18.2.01cor 33486653

[B16] Cuxac C. (1985). “Esquisse d’une typologie des langues des signes,” in *Autour de La Langue Des Signes*, ed. Cuxac C. (Paris: Université René Descartes), 35–60.

[B17] Cuxac C. (1996). *Fonctions et Structures de l’iconicité des Langues des Signes.* Ph. D. thesis. Paris: University of Paris.

[B18] Cuxac C. (1999). “The expression of spatial relations and the spatialization of semantic representations in French Sign Language,” in *Language Diversity and Cognitive Representations*, eds Fuchs C. Robert S. (Amsterdam: John Benjamins Publishing Company), 123–142.

[B19] Cuxac C. (2000). *La Langue des Signes Française: Les voies de l’iconicité.* Paris: Ophrys.

[B20] Cuxac C. Sallandre M. (2007). “Iconicity and arbitrariness in French Sign Language: highly Iconic Structures, degenerated iconicity and diagrammatic iconicity,” in *Verbal and Signed Languages: Comparing Structures, Constructs and Methodologies*, eds Pizutto E. Pietrandrea P. Simone R. (Berlin: Mouton de Gruyter), 13–33.

[B21] De Beuzeville L. Johnston T. Schembri A. (2009). The use of space with indicating verbs in Auslan: a corpus-based investigation. *Sign Lang. Linguist.* 12 53–82. 10.1075/sll.12.1.03deb 33486653

[B22] DeMatteo A. (1977). “Visual imagery and visual analogues in American Sign Language,” in *On the other Hand: New Perspectives on American Sign Language*, ed. Friedman L. (New York, NY: Academic Press), 215–236.

[B23] Dingemanse M. (2011). *The Meaning and Use of Ideophones in Siwu.* Ph. D. thesis. Nijmegen: Radboud University Nijmegen.

[B24] Dingemanse M. (2013). Ideophones and gesture in everyday speech. *Gesture* 13 143–165. 10.1075/gest.13.2.02.din 33486653

[B25] Dingemanse M. (2014). Making new ideophones in Siwu: creative depiction in conversation. *Pragmat. Soc.* 5 384–405.

[B26] Dingemanse M. (2015). Ideophones and reduplication: depiction, description, and the interpretation of repeated talk in discourse. *Stud. Lang.* 39 946–970. 10.1075/sl.39.4.05din 33486653

[B27] Dingemanse M. (2017). Expressiveness and system integration: on the typology of ideophones, with special reference to Siwu. *STUF Lang. Typol. Universals* 70:18.

[B28] Dudis P. (2004). Body partitioning and real space blends. *Cognit. Linguist.* 15 223–238.

[B29] Emmorey K. (1999). “Do Signers Gesture?,” in *Gesture, Speech and Sign*, eds Messing L. Campbell R. (Oxford: Oxford University Press), 133–159.

[B30] Enfield N. (2009). *The Anatomy of Meaning: Speech, Gesture and Composite Utterances.* Cambridge: Cambridge University Press.

[B31] Engberg-Pedersen E. (1993). *Space in Danish Sign Language: The Semantics and Morphosyntax of the Use of Space in a Visual Language.* Hamburg: Signum Verlag.

[B32] Engberg-Pedersen E. (2015). Perspective in signed discourse: the priviliged status of the signer‘s locus and eye gaze. *Open Linguist.* 1 411–431.

[B33] Fauconnier G. (1985). *Mental Spaces: Aspects of Meaning Construction in Natural Language.* Cambridge: MIT Press.

[B34] Fauconnier G. (1997). *Mappings in Thought and Language.* Cambridge: Cambridge University Press.

[B35] Fauconnier G. Turner M. (1996). “Blending as a Central Process of Grammar,” in *Conceptual Structure, Discourse, and Language*, ed. Goldberg A. (Stanford CA: CSLI), 113–130.

[B36] Fenlon J. Schembri A. Cormier K. (2018). Modification of indicating verbs in British Sign Language: a corpus-based study. *Language* 94 84–118.

[B37] Ferrara L. (2012). *A Grammar of Depiction: Exploring Gesture and Language in Australian Sign Language (Auslan).* Ph. D. thesis. Sydney: Macquarie University.

[B38] Ferrara L. Halvorsen R. (2017). Depicting and describing with iconic signs in Norwegian Sign Language. *Gesture* 16 371–395.

[B39] Ferrara L. Hodge G. (2018). Language as Description, Indication and Depiction. *Front. Psychol.* 9:716. 10.3389/fpsyg.2018.00716 29875712 PMC5974176

[B40] Ferrara L. Johnston T. (2012). “Enactment in discourse: conceptualization through language and gesture,” in *Paper Presented at the Language, Culture, and Mind (LCM) 5 conference 27-29 June, Universidade Católica Portuguesa, Lisbon, Portugal*, (Lisbon: Universidade Catoìlica Portuguesa).

[B41] Ferrara L. Johnston T. (2014). Elaborating Who’s what: a study of constructed action and clause structure in auslan (Australian Sign Language). *Austral. J. Linguist.* 2 193–215.

[B42] Friedman L. (1975). Space, Time and person reference in American Sign Language. *Language* 51 940–961.

[B43] Frishberg N. (1975). Arbitrariness and iconicity: historical change in American Sign Language. *Language* 51 696–719.

[B44] Garcia B. Sallandre M. (2019). “The Semiological Approach. What about the non-conventional units in sign language discourse,” in *Workshop EURASIGN Research Network, 5-6 April 2019*, (Sweden: EURASIGN).

[B45] Garcia B. Sallandre M. (2020). Contribution of the semiological approach to deixis–anaphora in sign language: the key role of eye-gaze. *Front. Psychol.* 11:2644. 10.3389/fpsyg.2020.583763 33240174 PMC7677344

[B46] Goodwin C. (1981). *Conversational Organization: Interaction between Speakers and Hearers.* New York, NY: Academic Press.

[B47] Hodge G. (2013). *Patterns from a Signed Language Corpus: Clause-Like Units in Auslan (Australian Sign Language).* Ph. D. thesis. Sydney: Macquirie University.

[B48] Hodge G. Ferrara L. (2014). “Showing the story: enactment as performance in Auslan narratives,” in *Selected Papers from the 44th Conference of the Australian Linguistic Society 2013*, eds Gawne L. Vaughan J. (Melbourne: University of Melbourne), 372–397.

[B49] Hsu H. (2021). When gesture “takes over”: speech-embedded nonverbal depictions in multimodal interaction. *Front. Psychol.* 11:552533. 10.3389/fpsyg.2020.552533 33643106 PMC7906077

[B50] Jantunen T. (2008). Fixed and Free: order of the verbal predicate and its core arguments in declarative transitive clauses in Finnish Sign Language. *SKY J. Linguist.* 21 83–123.

[B51] Jantunen T. (2017). Constructed Action, the Clause and the Nature of Syntax in Finnish Sign Language. *Open Linguist.* 3 65–85.

[B52] Janzen T. (2012). “Lexicalization and grammaticalization,” in *Sign Languages*, eds Pfau R. Steinbach M. Woll B. (Berlin: Mouton De Gruyter), 816–841.

[B53] Janzen T. (2017). Composite utterances in a signed language: topic constructions and perspective-taking in ASL. *Cognit. Linguist.* 28 511–538. 10.1515/cog-2016-0121

[B54] Johnston T. (1991). Spatial syntax and spatial semantics in the inflection of signs for the marking of person and location in Auslan. *Int. J. Sign Lang. Linguist.* 2 29–62.

[B55] Johnston T. (1996). “Function and medium in the forms of linguistic expression found in a sign language,” in *Modality and Structure in Signed and Spoken Languages*, eds Edmonson W. H. Wilbur R. (Cambridge: Cambridge University Press), 199–224.

[B56] Johnston T. (2011/2013). *Auslan Corpus Annotation Guidelines: Transcription and Annotation in the Creation of Signed Language Corpora.* Sydney: Macquarie University.

[B57] Johnston T. (2013). Towards a comparative semiotics of pointing actions in signed and spoken languages. *Gesture* 13 109–142. 10.1075/gest.13.2.01joh 33486653

[B58] Johnston T. (2019). Clause constituents, arguments and the question of grammatical relations in Auslan (Australian Sign Language): a corpus-based study. *Stud. Lang.* 43 944–998.

[B59] Johnston T. Ferrara L. (2012). “Lexicalization in signed languages: when is an idiom not an idiom?,” in *Selected Papers from United Kingdom Cognitive Linguistics Association Meetings*, Vol. 1, ed. Hart C. (Hertfordshire: UK Cognitive Linguistics Association), 229–248.

[B60] Johnston T. Schembri A. (1999). On defining a lexeme in a Signed Language. *Sign Lang. Linguist.* 2 115–185.

[B61] Johnston T. Schembri A. (2007). *Australian Sign Language: An Introduction to Sign Language Linguistics.* Cambridge: Cambridge University Press.

[B62] Johnston T. Schembri A. (2010). Variation, lexicalization and grammaticalization in signed languages. *Lang. Soc.* 1, 19–35. 10.3917/ls.131.0019 18052372

[B63] Johnston T. Vermeerbergen M. Schembri A. Leeson L. (2007). ““Real data are messy”: considering cross-linguistic analysis of constituent ordering in Auslan, VGT, ISL,” in *Visible Variation: Comparative Studies on Sign Language Structure*, eds Perniss P. Pfau R. Steinbach M. (Berlin: Mouton de Gruyter), 163–205.

[B64] Kegl J. Schley S. (1986). “When is a Classifier No Longer a Classifier?,” in *Proceedings of the Twelfth Annual Meeting of the Berkeley Linguistics Society*, (Berkeley: Berkeley Linguistics Society), 425–441. 10.3765/bls.v12i0.1881

[B65] Kegl J. Wilbur R. (1976). “When does structure stop and style begin? Syntax, morphology, and phonology vs. stylistic variation in ASL,” in *CLS 12*, (Chicago: Chicago University Press).

[B66] Kendon A. (2004). *Gesture: Visible Action as Utterance.* Cambridge: Cambridge University Press, 10.1017/CBO9780511807572

[B67] Lentz E. M. (1986). “Teaching Role Shifting,” in *Proceedings of the Fourth National Symposium on Sign Language Research and Teaching, Sign*, ed. Padden C. (Siler Spring, MD: NAD), 58–69.

[B68] Lepic R. Occhino C. (2018). “A Construction Morphology Approach to Sign Language Analysis,” in *The Construction of Words: Advances in Construction Morphology*, ed. Booij G. (New York, NY: Springer), 141–172.

[B69] Liddell S. K. (1980). *American Sign Language syntax.* Mouton: The Hague.

[B70] Liddell S. K. (1990). “Four Functions of a locus: reexamining the structure of space in ASL,” in *Sign Language Research: Theoretical Issues*, ed. Lucas C. (Washington DC: Gallaudet University Press), 176–198.

[B71] Liddell S. K. (2000). “Indicating verbs and pronouns: pointing away from agreement,” in *The signs of language revisited: An anthology to honor Ursula Bellugi and Edward Klima*, eds Emmorey K. Lane H. (Mahwah NJ: Lawrence Erlbaum), 303–320.

[B72] Liddell S. K. (2003). *Grammar, Gesture and Meaning in American Sign Language.* Cambridge: Cambridge University Press.

[B73] Liddell S. K. Johnson R. (1987). “An analysis of spatial locative predicates in American Sign Language,” in *Paper presented at the 4*^th^* International Symposium on Sign Language Research, 15-19 July*, (Salamanca: ICSLA).

[B74] Liddell S. K. Metzger M. (1998). Gesture in sign language discourse. *J. Pragmat.* 30 657–697. 10.1016/S0378-2166(98)00061-7

[B75] Loew R. C. (1984). *Roles and Reference in American Sign Language: A Developmental Perspective.* Ph. D. thesis. Minneapolis: University of Minnesota.

[B76] Mandel M. (1977). “Iconic devices in american sign language,” in *On the Other Hand: New Perspectives on American Sign Language*, ed. Lynn A. F. (New York, NY: Academic Press), 57–107.

[B77] Mayer M. (1969). *Frog, Where Are You? SEQUEL to a Boy, a Dog and a Frog.* New York, NY: Dial Books for Young Readers.

[B78] McNeill D. (1992). *Hand and Mind: What Gesture Reveals About Thought.* Chicago, CHI: University of Chicago Press.

[B79] Metzger M. (1995). “Constructed dialogue and constructed action in American Sign Language,” in *Sociolinguistics in Deaf Communities*, ed. Lucas C. (Washington, D.C: Gallaudet University Press), 255–271.

[B80] Mulrooney K. (2006). *The Structure of Personal Narratives in American Sign Language.* Washington, D.C.: Gallaudet University Press.

[B81] Padden C. (1986). “Verbs and role shifting in American Sign Language,” in *Proceedings of the Fourth National Symposium on Sign Language Research and Teaching, Sign*, ed. Paden C. (Silver Spring, MD: NAD), 44–57.

[B82] Peirce C. (1894). “What is a sign?,” in *The Essential Peirce Volume 2: Selected Philosophical Writings (1893-1913)*, eds Houser N. Eller J. R. De Tienne A. Lewis A. C. Clark C. L. Davis D. B. (Bloomington, IN: Indiana University Press), 4–10.

[B83] Peirce C. S. (1955). *Philosophical Writings of Peirce.* New York, NY: Dover Publications.

[B84] Perniss P. (2007). *Space and Iconicity in German Sign Language.* Ph. D. thesis. Nijmegen: Radboud University.

[B85] Puupponen A. (2019). Towards understanding nonmanuality: a semiotic treatment of singers’ head movements. *Glossa J. General Linguist.* 4:39. 10.5334/gjgl.709

[B86] Quer J. (2018). On categorizing types of role shift in Sign languages. *Theoretic. Linguist.* 44 277–282.

[B87] Quinto-Pozos D. (2007). Can constructed action be considered obligatory? *Lingua* 117 1285–1314.

[B88] Sallandre M. (2003). *Les unités du discourse en Langue des Signes Française: Tentative de Catégorisation dans le Cadre d’une Grammaire de l’iconicité.* Ph. D. thesis. Paris: University Paris.

[B89] Sallandre M. (2007). “Simultaneity in French Sign Language discourse,” in *Simultaneity in Signed Languages: Form and function*, eds Vermeerbergen M. Leeson L. Crasborn O. (Amsterdam: John Benjamins Publishing Company), 103–125.

[B90] Schembri A. (2003). “Rethinking classifier constructions in sign languages,” in *Perspectives on Classifier Constructions in Sign Languages*, ed. Emmorey K. (New York, NY: Psychology Press).

[B91] Schick B. (1990). Classifier predicates in American Sign Language. *Int. J. Sign Linguist.* 1 15–40.

[B92] Shaffer B. Janzen T. (2002). “Gesture as the substrate in the process of ASL grammaticalization,” in *Modality and Structure in Signed and Spoken Languages*, eds Meier In R. P. Cormier K. Quinto-Pozos D. (Cambridge: Cambridge University Press), 199–223. 10.1017/CBO9780511486777.010

[B93] Slonimska A. Özyürek A. Capirci O. (2021). Using depiction for efficient communication in LIS (Italian Sign Language). *Lang. Cognit.* 13 367–396. 10.1017/langcog.2021.7

[B94] Stec K. Huiskes M. Redeker G. (2016). Multimodal quotation: role shift practices in spoken narratives. *J. Pragmat.* 104 1–17. 10.1016/j.pragma.2016.07.008

[B95] Streeck J. (1993). Gesture as communication I: its coordination with gaze and speech. *Commun. Monogr.* 60 275–299.

[B96] Stukenbrock A. (2014). Pointing to an ‘empty’ space: deixis am Phantasma in face-to-face interaction. *J. Pragmat.* 74 70–93.

[B97] Supalla T. (1978). “Morphology of verbs of location and motion in American Sign Language,” in *Proceedings of the 2nd National Symposium on Sign Language Research and Teaching*, eds Caccamise C. Hicks D. (Coronado, CA: National Association of the Deaf), 27–46.

[B98] Supalla T. (1986). “The classifier system in American Sign Language,” in *Noun Classification and Categorization*, ed. Craig C. (Philadelphia: John Benjamins), 181–214.

[B99] Supalla T. (1990). “Serial verbs of motion in ASL,” in *Theoretical Issues in Sign Language Research*, eds Fischer S. Siple P. (Chicago, IL: University of Chicago Press), 127–152.

[B100] Takkinen R. (1996). “Classifiers in a sign language dictionary,” in *Paper presented at the Fifth International Conference on Theoretical Issues in Sign Language Research*, (Montreal: Sign Language Research).

[B101] Tannen D. (1986). *Talking Voices: Repetition, Dialogue an Imagery in Conversational Discourse.* Cambridge: Cambridge University Press.

[B102] Van Herreweghe M. (1995). *De Vlaams-Belgische gebarentaal: een eerste verkenning.* Gent: Academia Press.

[B103] Van Herreweghe M. Vermeerbergen M. (2003). “Het Opsporen en Invullen van ‘Gaten’ in het Lexicon van de Vlaamse Gebarentaal,” in *Invulling Van Hiaten in de Vlaamse Gebarentaal: Aardrijkskunde en Geschiedenis*, eds De Weerdt K. Rogiest M. (Gent: Cultuur voor Doven vzw), 2–10.

[B104] Van Herreweghe M. Vermeerbergen M. Demey E. De Durpel H. Nyffels H. Verstraete S. (2015). *Het Corpus VGT. Een digitaal open access corpus van video’s en annotaties van Vlaamse Gebarentaal, ontwikkeld aan de Universiteit Gent i.s.m.* Leuven: KU Leuven.

[B105] Vermeerbergen M. (1996). *Rood kool tien persoon. Morfo-syntactische aspecten van de Vlaams-Belgische Gebarentaal.* Ph. D. thesis. Brussel: Vrije Universiteit Brussel.

[B106] Vermeerbergen M. (1997). *Grammaticale aspecten van de Vlaamse Gebarentaal.* Destelbergen: Cultuur voor Doven vzw.

[B107] Vermeerbergen M. (2006). Past and current trends in sign language research. *Lang. Commun.* 26 168–192. 10.1016/j.langcom.2005.10.004

[B108] Vermeerbergen M. (2013). *Vast en productief lexicon.* [Unpublished manuscript].

[B109] Vermeerbergen M. (2014). “When signers and speakers construct action, do they (also/always) shift role?,” in *Talk presented at Mapping Multimodal Dialogue 3 (MaMuD), KU Leuven, Leuven, 21 November 2014*, (Leuven: KU Leuven).

[B110] Vermeerbergen M. (2016). “When there is little difference between the frozen sign) TAP, (the classifier sign) “open-flask” and CA: “open-jar-with-great-difficulty”: defining and describing the “productive lexicon” in signed languages,” in *Talk Presented at Sign Pop-Up*, (Nijmegen: Radboud University Nijmegen).

[B111] Vermeerbergen M. Demey E. (2007). “Sign + Gesture = Speech + Gesture? Comparing aspects of Simultaneity in Flemish Sign Language to instances of concurrent speech and gesture,” in *Simultaneity in Signed Languages: Form and Function*, eds Vermeerbergen M. Leeson L. Crasborn O. (Amsterdam: John Benjamins Publishing Company), 257–282.

[B112] Wallin L. (1990). “Polymorphemic predicates in Swedish Sign Language,” in *Sign Language Research: Theoretical Issues*, ed. Lucas C. (Washington DC: Gallaudet University Press).

[B113] Winston E. (1991). Spatial Referencing and Cohesion in an American Sign Language Text. *Sign Lang. Stud.* 73 397–410.

[B114] Winston E. (1992). “Space and involvement in an American Sign Language lecture,” in *Expanding Horizons: Proceedings of the twelfth national convention of the Registry of Interpreters for the Deaf*, ed. Pland-Moeller J. (Silver Spring, MD: RID publications), 93–105.

